# Loss of function mutation of the Rapid Alkalinization Factor (RALF1)-like peptide in the dandelion *Taraxacum koksaghyz* entails a high-biomass taproot phenotype

**DOI:** 10.1371/journal.pone.0217454

**Published:** 2019-05-24

**Authors:** Annika Wieghaus, Dirk Prüfer, Christian Schulze Gronover

**Affiliations:** 1 Institute of Plant Biology and Biotechnology, University of Muenster, Münster, Germany; 2 Fraunhofer Institute for Molecular Biology and Applied Ecology (IME), Münster, Germany; College of Agricultural Sciences, UNITED STATES

## Abstract

The Russian dandelion (*Taraxacum koksaghyz*) is a promising source of inulin and natural rubber because large amounts of both feedstocks can be extracted from its roots. However, the domestication of *T*. *koksaghyz* requires the development of stable agronomic traits such as higher yields of inulin and natural rubber, a higher root biomass, and an agronomically preferable root morphology which is more suitable for cultivation and harvesting. *Arabidopsis thaliana* Rapid Alkalinisation Factor 1 (RALF1) has been shown to suppress root growth. We identified the *T*. *koksaghyz* orthologue TkRALF-like 1 and knocked out the corresponding gene (*TkRALFL1*) using the CRISPR/Cas9 system to determine its impact on root morphology, biomass, and inulin and natural rubber yields. The *TkRALFL1* knockout lines more frequently developed a taproot phenotype which is easier to cultivate and harvest, as well as a higher root biomass and greater yields of both inulin and natural rubber. The *TkRALFL1* gene could therefore be suitable as a genetic marker to support the breeding of profitable new dandelion varieties with improved agronomic traits. To our knowledge, this is the first study addressing the root system of *T*. *koksaghyz* to enhance the agronomic performance.

## Introduction

The Russian dandelion (*Taraxacum koksaghyz*) is a sexually reproducing, self-incompatible, perennial plant species in the family Asteraceae [1], renowned for its rapid growth and climatic versatility. *T*. *koksaghyz* has recently emerged as a promising industrial feedstock for temperate regions because of its ability to produce and store significant amounts of natural rubber and inulin in its roots, although complete domestication is required before it can be adopted as an industrial crop [2,3].

Research on the Russian dandelion has focused on the manipulation of natural rubber and inulin metabolism. Natural rubber is an economically important biopolymer which is currently sourced mainly from the rubber tree *Hevea brasiliensis* [4]. However, the search for alternative sources has been encouraged by the growing demand for natural rubber and the disadvantages of *H*. *brasiliensis*, including slow growth, strict climate requirements, the susceptibility of monoculture plantations to pathogens, and the costs of transporting the raw material from current geographically limited production sites [2,5,6]. In *T*. *koksaghyz*, natural rubber is mainly synthesized and stored in the roots, with yields of up to 15% dry weight [2]. The roots also accumulate the storage carbohydrate inulin, with yields of up to 50% dry weight [7]. Inulin is used as a food additive but is also a feedstock for bioethanol production [8,9]. It is currently extracted from chicory roots [10].

Before *T*. *koksaghyz* can be developed as an alternative source of natural rubber and inulin, its agronomic performance must be modified to enhance its productivity and the ease of cultivation and harvesting. Field trials covering multiple growing seasons have provided data concerning sowing time, cultivation time and conditions, and the optimum time of harvest, aiming to increase the yields of natural rubber and inulin [6,11,12]. However, little has yet been done to address factors such as root morphology, which determines the ease of harvesting and facilitates the recovery of raw materials from root tissue.

Plant root growth and development are influenced by many pathways and physiological processes including short-distance cell-to-cell communication. This involves small signalling peptides (peptide hormones) which are secreted into the apoplast, where they are involved in cellular signaling and the regulation of developmental processes [13–15]. The Rapid Alkalinization Factor (RALF) family comprises a group of cysteine-rich peptides found throughout the plant kingdom [16]. The different members of the *Arabidopsis thaliana RALF* family show various tissue-specific and developmentally regulated expression profiles [17–20] including the root-specific expression of *AtRALF1* [21]. The overexpression of *AtRALF1* and treatment with exogenous RALF peptide reduced the extent of root growth in *A*. *thaliana* seedlings, whereas the downregulation of *AtRALF1* promoted root elongation [21–24]. Similar results were observed in seedlings of the wild tobacco species *Nicotiana attenuata* [25] and in tomato [26]. The analysis of *A*. *thaliana* roots by microscopy revealed that the differences in growth between plants overexpressing *AtRALF1* and the corresponding knockdown lines reflected differences in root cell size [24]. RALF pro-peptides have an N-terminal signal peptide for secretion [26] which is cleaved at an RRXL motif by a serine protease during maturation [23]. The mature peptide contains four highly conserved cysteine residues, which are considered necessary for the folding of the processed peptide [26,27], and a functionally critical conserved YISY motif [28]. AtRALF1 is thought to regulate cell expansion via cell-to-cell communication, which involves downstream mechanisms such as Ca^2+^ and MAP kinase signalling, and the modulation of pH in the apoplast [27]. The latter requires AtRALF1 to bind the recently identified receptor FERONIA, triggering the phosphorylation of a cell membrane H^+^ATPase which increases the apoplast pH [21]. This initiates a signaling cascade that inhibits cell elongation and thus reduces root growth, whereas increased root growth occurs when the RALF peptide is depleted or missing [25–27,29]. Given the properties of *A*. *thaliana* RALF1, a *T*. *koksaghyz* homologue could potentially be used to improve agronomic qualities such as root biomass, and inulin and rubber yield.

Here we show that a lack of a RALF1-like peptide in *T*. *koksaghyz* positively influences the root system, yielding plants with an agronomically preferable taproot morphology, greater root biomass, and higher yields of inulin and natural rubber. Furthermore, our data suggest that *T*. *koksaghyz RALFL1* could be used as a molecular marker for the development of *T*. *koksaghyz* varieties with enhanced agronomic performance using modern breeding strategies, which is essential for the establishment of a commercially viable crop.

## Results

### Identification and characterization of *T*. *koksaghyz* RALFL1

**Fig 1:**
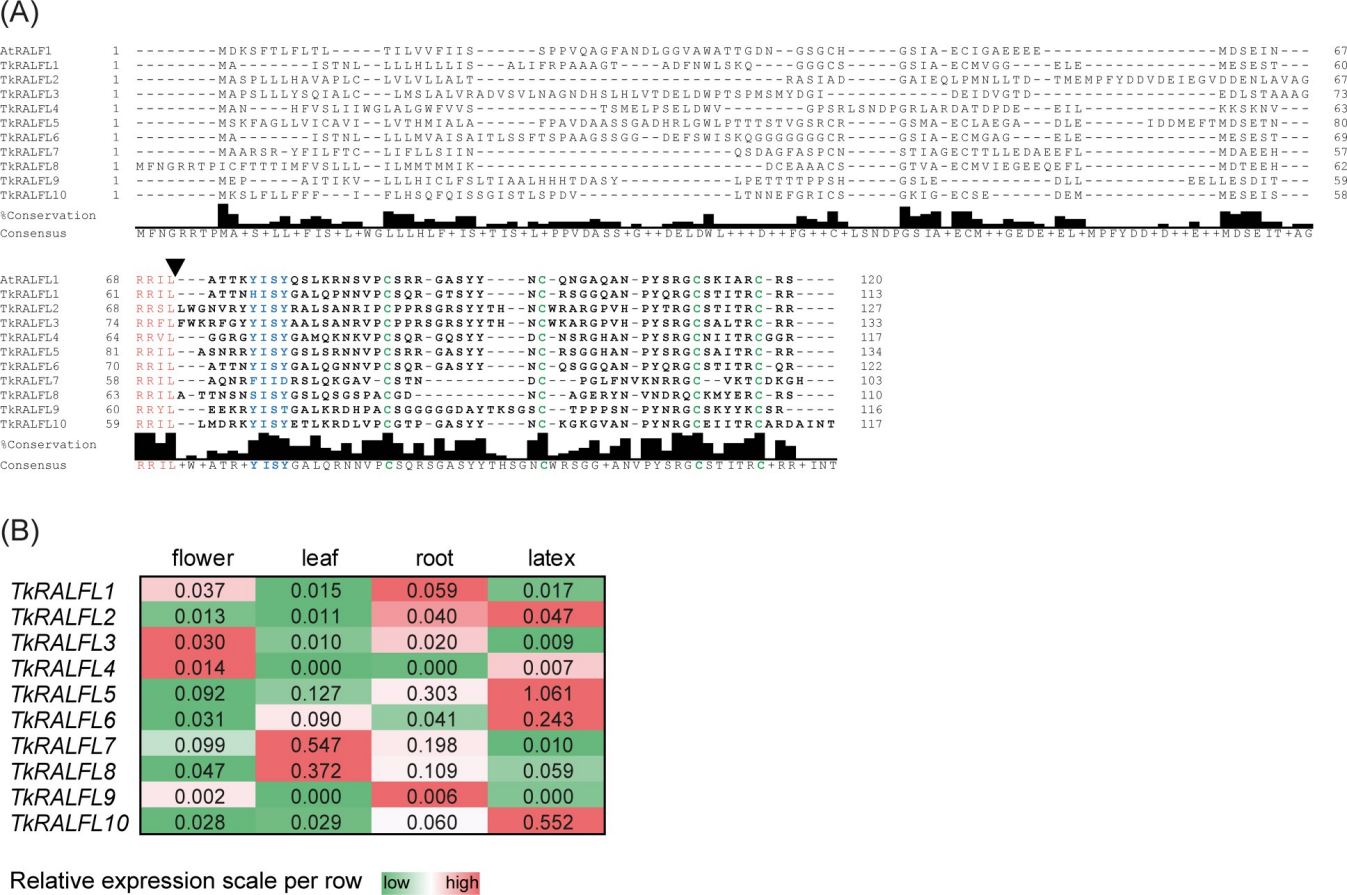
Multiple sequence alignment of AtRALF1 and TkRALFL1–TkRALFL10, and the spatial expression profiles of *TkRALFL1–TkRALFL10*. (A) The alignment of AtRALF1 (AEE27494.1) and the deduced amino acid sequences of TkRALF1–TkRALFL10 was carried out using MegAlign Pro software (DNASTAR Inc., Madison, Wisconsin, USA) with the MUSCLE algorithm, and was edited with JalView software [32]. The mature peptide is shown in bold letters, the RRXL motif is highlighted in red, the arrowhead depicts the cleavage site behind the RRXL motif, the YISY motif is highlighted in blue, and the four conserved cysteine residues are highlighted in green. The consensus sequence is shown below the alignment; additionally the height of the black columns above indicates the percentage of the modal residue per column (labelled with %Conservation). (B) The spatial expression of *TkRALFL1–TkRALFL10* in 12-week-old wild-type *T*. *koksaghyz* plants was determined by qPCR. The mRNA levels were normalized using the reference genes elongation factor 1α (*Tkef1α*) and ribosomal protein L27 (*TkRP*). The expression levels are coloured by relative scale per row in order to highlight differences in gene expression of the respective *TkRALFL* gene among the different tissues, with green depicting a low expression level and red a high expression level (n = 8–12 plants per tissue).

We screened the available body of *T*. *koksaghyz* sequence data using known RALF sequences including *A*. *thaliana* RALF1 (AEE27494.1) which is known to play a key role in root development. We identified 10 *RALF*-like sequences, which we named *TkRALFL1*–*TkRALFL10*. Each comprised a single exon, in agreement with recently published genome sequence data [30]. *In silico* analysis revealed that all 10 sequences encoded small peptides of 103–134 amino acids (Fig 1A). The peptides contained features typical of the RALF family [27], including an N-terminal signal peptide of varying length. All 10 TkRALFL peptides featured an RRXL motif with a dibasic amino acid sequence as a protease recognition site [22,23]. The mature peptides included four highly conserved cysteine residues [26] as well as the YISY motif with different substitutions at amino acid positions that are not necessary for RALF activity [28]. Multiple sequence alignment of all 10 TkRALFL amino acid sequences and AtRALF1 using MUSCLE (Fig 1A) revealed an overall higher percentage of conserved amino acids within the mature peptides (illustrated by the black columns in Fig 1A below the alignment, above the consensus sequence), confirming the highly conserved C-terminus described for many RALF peptides [31]. The distance matrix of the alignment (S1 Table) revealed three TkRALFL peptides that were closely related to AtRALFL1, with 62% identity in each case (TkRALFL1, TkRALFL5 and TkRALFL6).

Given that *AtRALF1* is prominently expressed in roots compared to other tissues [21], we investigated the spatial expression of all *TkRALFL* transcripts by qPCR (Figs 1B and S1). The *TkRALFL* genes showed diverse expression profiles encompassing different tissues. We found that *TkRALFL1* and *TkRALFL9* were more strongly expressed in the roots than the flower, leaf or latex, but *TkRALFL9* was expressed at very low levels in all tissues. *TkRALFL5* and *TkRALFL6*, which also showed a high percent identity to *AtRALF1*, were strongly expressed in the latex but not in root tissues. Some genes including *TkRALFL5* showed a higher expression in roots than TkRALFL1, but did not exhibit a predominant expression in the roots compared to the other tissues. Therefore, *TkRALFL1* emerged as most promising candidate for a *T*. *koksaghyz* orthologue of *AtRALF1* and was selected for further investigation.

### Exogenous TkRALFL1 inhibits primary root growth in *T*. *koksaghyz* seedlings

**Fig 2:**
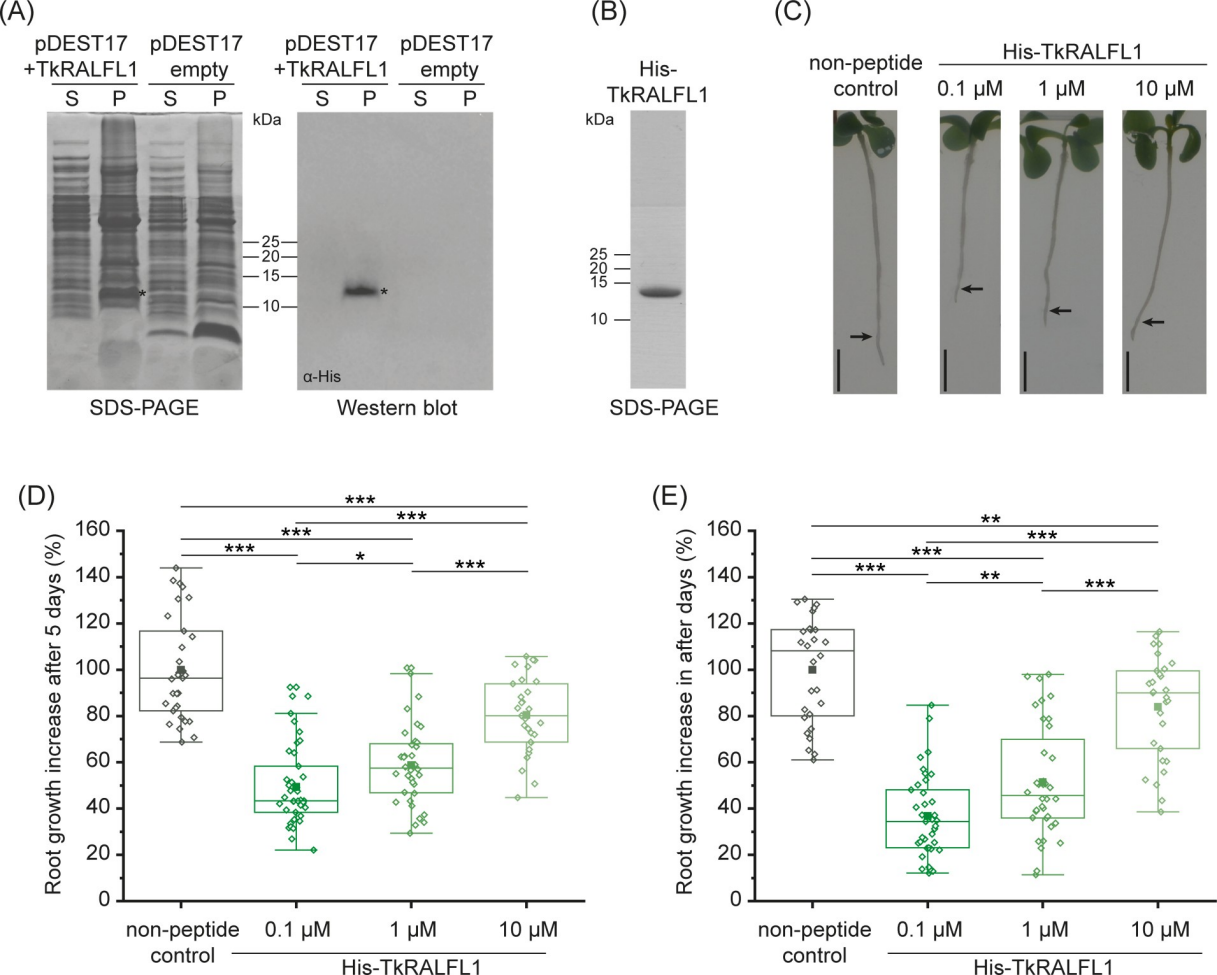
Effect of TkRALF1 produced in *E*. *coli* on primary root growth. (A) SDS-PAGE and subsequent western blot analysis (with a His-tag specific antibody) of total protein from *E*. *coli* BL21 transformed with either the vector containing His-TkRALFL1 or the empty vector control. For western blot analysis, proteins were transferred to a PVDF membrane and probed using a His-tag specific antibody conjugated to horseradish peroxidase. S, soluble protein phase; P, insoluble protein phase; SDS-PAGE stained with Coomassie Brilliant Blue. The asterisk indicates the additional band in the insoluble phase of *E*. *coli* transformed with the expression vector containing His-TkRALFL1. (B) SDS-PAGE analysis of the TkRALFL1 peptide after Ni-NTA affinity purification and subsequent dialysis in order to promote the refolding of the peptide. Gel stained with PageBlue Protein Staining Solution. (C) Representative *T*. *koksaghyz* wild-type seedlings from the root growth assay 5 days after transfer to the medium containing different concentrations of TkRALFL1 peptide, that are indicated above the images. Pure buffer was used as non-peptide control. The arrow indicates the position of the root tip at the time of transfer; scale bar = 5 mm. (D) Root growth increase of the primary root of *T*. *koksaghyz* wild-type seedlings after treatment for 5 days with different concentrations of exogenous mature TkRALFL1 peptide dissolved in buffer (0.1% (v/v) formic acid). Values were normalized to the mean growth increase of seedlings treated with pure buffer as non-peptide control. The box delimits values from the 25^th^ to the 75^th^ percentile of the dataset, the horizontal line in the box represents the median of the data, the filled square represents mean value, and the lower and upper whiskers represent obtained values that differ least from 25^th^ percentile –1.5*IQR or 75^th^ percentile +1.5*IQR, respectively; n = 28–39, normal distribution proved by Kolmogorov-Smirnov test, statistical significance proved by two-tailed *t*-test, *p<0.05, **p<0.01, ***p<0.001. (E) Root growth increase of the primary root of *T*. *koksaghyz* wild-type seedlings after treatment for 10 days as described in Fig 2D. Box plot as described above.

Exogenous RALF1 was previously shown to inhibit root growth in *A*. *thaliana* seedlings [20,26]. To test whether TkRALFL1 functions in a similar manner, we expressed the mature peptide with an N-terminal His-tag (estimated molecular mass 8.3 kDa) in the *Escherichia coli* strain BL21. SDS-PAGE analysis revealed a ~13-kDa band in the insoluble protein fraction that was not present in the untransformed control cells. Subsequent western blot analysis using a His-tag-specific antibody confirmed the presence of the His-tagged peptide (Fig 2A). Analysis of the isolated peptide by mass spectrometry clearly identified the TkRALFL1-specific peptide sequences covering 61.64% of the expressed His-TkRALFL1 sequence (S2 Table). We purified the peptide by Ni-NTA affinity chromatography under denaturing conditions, followed by dialysis against 0.1% formic acid to remove the denaturing agent and promote refolding of the peptide, and finally freeze-drying. SDS-PAGE analysis of the peptide dissolved in 0.1% formic acid revealed a single band of the expected size (Fig 2B).

The recombinant protein was treated with pepsin since a cleavage site for pepsin at a pH of 1.3 was present (predicted by ExPASy PeptideCutter [33]) within the mature TkRALFL1 sequence, yielding the shortened peptide TkRALFL1(11–49) (S2A and S2B Fig). TkRALFL1(11–49) lacks the HISY motif that is described to be essential for activity of RALF peptides and is equivalent to AtRALF(9–49), which served as a common control for root growth assays [26,28]. TkRALFL1(11–49) was applied in a first root growth assay. Seven-day-old wild-type *T*. *koksaghyz* seedlings were transferred either to medium containing 0.1 μM TkRALFL1(11–49) or to control medium. The growth increase of the primary roots did not show any difference compared to the non-peptide control (S2C Fig).

The purified recombinant peptide was then used in a root growth assay testing different concentrations of TkRALFL1 in the medium. Wild-type *T*. *koksaghyz* seedlings were transferred either to medium containing 0.1, 1 or 10 μM TkRALFL1 or to control medium. After 5 days, the increase in primary root length was measured and revealed a dose-dependent response of the roots. When transferred to medium containing 0.1 μM TkRALFL1, the root growth was inhibited by ~51% compared to seedlings grown on non-peptide control plates. 1 μM and 10 μM TkRALFL1 inhibited root growth by ~41% and 20%, respectively. (Fig 2C and 2D). The growth inhibition was not a transient effect and still visible after 10 days of treatment in the same extent (Fig 2E).

**Fig 3:**
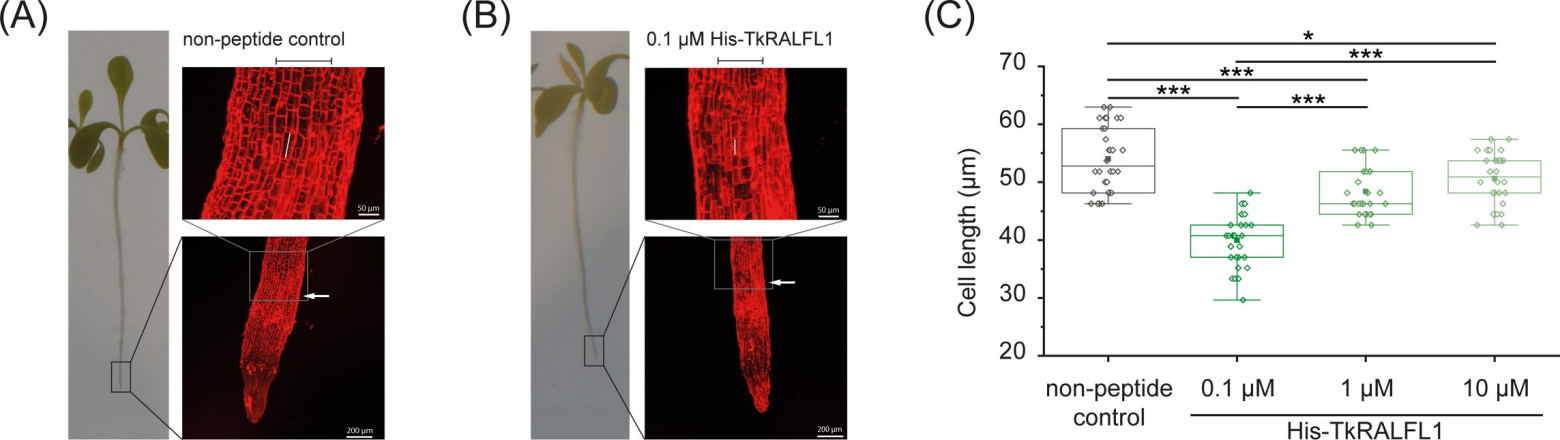
Detailed analysis of primary root cell length after treatment with TkRALFL1. (A) Root tip image acquired by CLSM of a representative seedling treated for 10 days with non-peptide control. Seedlings were stained with propidium iodide (0.1 mM). The white arrow indicates the distance of 1 mm from the very tip; scale bar as indicated in the respective images; the black bar above the magnified segment indicates the most inner six rows of rhizodermal cells measured for evaluation of the cell length, one cell is exemplarily marked by a white bar. (B) Root tip image of a representative seedling treated for 10 days with 0.1 μM His-TkRALFL1 as described in Fig 3A. (C) Cell length of rhizodermal cells from root tips of seedlings treated for 10 days with different concentrations of TkRALFL1 as indicated. n = 30, normal distribution proved by Kolmogorov-Smirnov test, statistical significance proved by two-tailed *t*-test, *p<0.05, ***p<0.001. Box plot as described in Fig 2.

Additionally, the cell length was measured in more detail using images of the root tips acquired by confocal laser scanning microscopy (Fig 3A and 3B). The evaluation of the rhizodermal cell length after 10 days of treatment with TkRALFL1 peptide revealed strongly reduced cell lengths for all tested concentrations, again in a dose-dependent manner (Fig 3C). The *in silico* analysis, spatial expression profile and root growth assays together provide convincing evidence that TkRALFL1 is involved in regulation of primary root growth in *T*. *koksaghyz*.

### CRISPR/Cas9 knockout of the *TkRALFL1* gene induces a taproot phenotype

**Fig 4:**
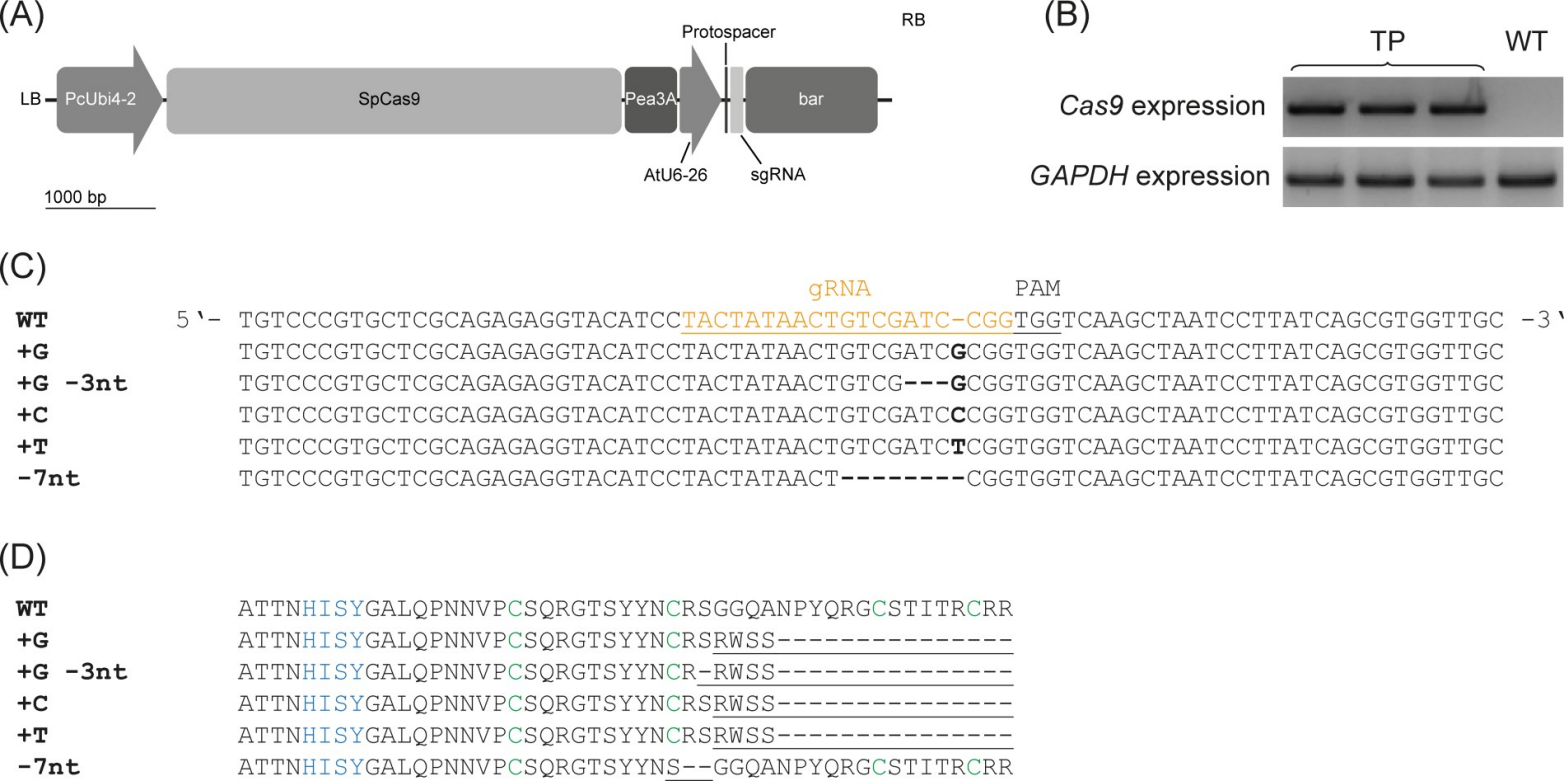
Mutation analysis of the TkRALFL1 CRISPR/Cas9 plants. (A) Schematic depiction of the TkRALFL1-CRISPR/Cas9 construct. PcUbi4-2, Ubiquitin4-2 promoter from *Petroselinum crispum*; SpCas9, Cas9 nuclease from *Streptococcus pyogenes*; Pea3A, *pea3A* terminator from *Pisum sativum*; AtU6-26, U6-26 promoter from *Arabidopsis thaliana*; sgRNA, single-guide RNA; *bar*, phosphinothricin resistance cassette from pFGC5941 vector; LB, left border; RB, right border. (B) Analysis of the *cas9* expression in the transgenic T0 plants and a wild-type control. The housekeeping gene *glycerine aldehyde-3-phosphate dehydrogenase* (*GAPDH*) was amplified as a control. TP, transgenic T0 plants; WT, wild-type control plant. (C) Nucleotide alignment of sequencing results from the T0 generation, showing the section around the CRISPR target site for different observed mutations. Sequences were obtained by amplification of the complete *TkRALFL1* sequence, followed by transfer to the TOPO sequencing vector. The protospacer adjacent motif (PAM) is underlined; the protospacer is underlined and orange; mutations are highlighted in bold letters or with dashes and are listed on the right side. (D) *In silico* analysis of the deduced amino acid sequences resulting from the mutations induced by CRISPR/Cas9, showing the mature peptide sequence. Sequences were aligned using MUSCLE algorithm. The YISY motif is highlighted in blue, the conserved cysteine residues are highlighted in green, and amino acids affected by the frameshift introduced by the mutations are underlined.

The *TkRALFL1* gene was targeted using CRISPR/Cas9. A protospacer sequence specific for *TkRALFL1* was designed and introduced into the CRISPR/Cas9 vector (Fig 4A). Transgenic *T*. *koksaghyz* plants carrying the sgRNA and *cas9* transgenes were produced using *Agrobacterium tumefaciens*, and *cas9* expression was confirmed in three T0 transformants (Fig 4B). Genome editing was confirmed in all three of these T0 plants by sequencing the complete *TkRALFL1* locus and by DNA fragment length analysis (FLA) to detect indels (Figs 4C and S3). In one plant, both *TkRALFL1* alleles were edited, with the insertion of one nucleotide in one allele (+1 nt) and an insertion of one nucleotide plus a deletion of three nucleotides (–2 nt in total) in the other allele. FLA confirmed the sequencing data, with one peak in the electropherogram shifted by +1 and the other by –2 compared to the wild-type electropherogram. The other two T0 plants contained one wild-type allele and one allele with an insertion of one nucleotide, but we also detected rare mutations such as the deletion of seven nucleotides. FLA in these two plants revealed two major peaks in the electropherogram, one at the wild-type position and one shifted by +1, as well as minor peaks shifted by more than –1. The presence of these minor peaks suggested the plants might be chimeric. Nevertheless, *in silico* analysis of the amino acid sequences deduced from the detected mutations predicted a premature stop codon or shortened sequence in all cases, causing the removal of the functionally-critical cysteine residues (Fig 4D). Therefore, all three T0 plants were used to produce homozygous progeny for further investigation.

The *TkRALFL* family is highly conserved, especially at the C-terminus, so we checked for the presence of off-target mutations in the other *TkRALFL* genes. Sequence alignment of the *TkRALFL1*-specific protospacer and the sequences of *TkRALFL1–TkRALFL10* using the BLAST algorithm [34] revealed a sequence identity of 91% between the protospacer and *TkRALFL6* (S4A Fig). The alignment, spanning 23 nucleotides, revealed two mismatches within the seed region of the protospacer, the complete accuracy of which is necessary for sgRNA binding and cleavage by Cas9 [35]. However, amplification and subsequent sequencing of *TkRALFL6* in the transgenic plants did not indicate any sequence alterations (S4B Fig). Sequence alignments with other *RALFL* genes showed only short stretches of similar nucleotides and were therefore ignored.

**Fig 5:**
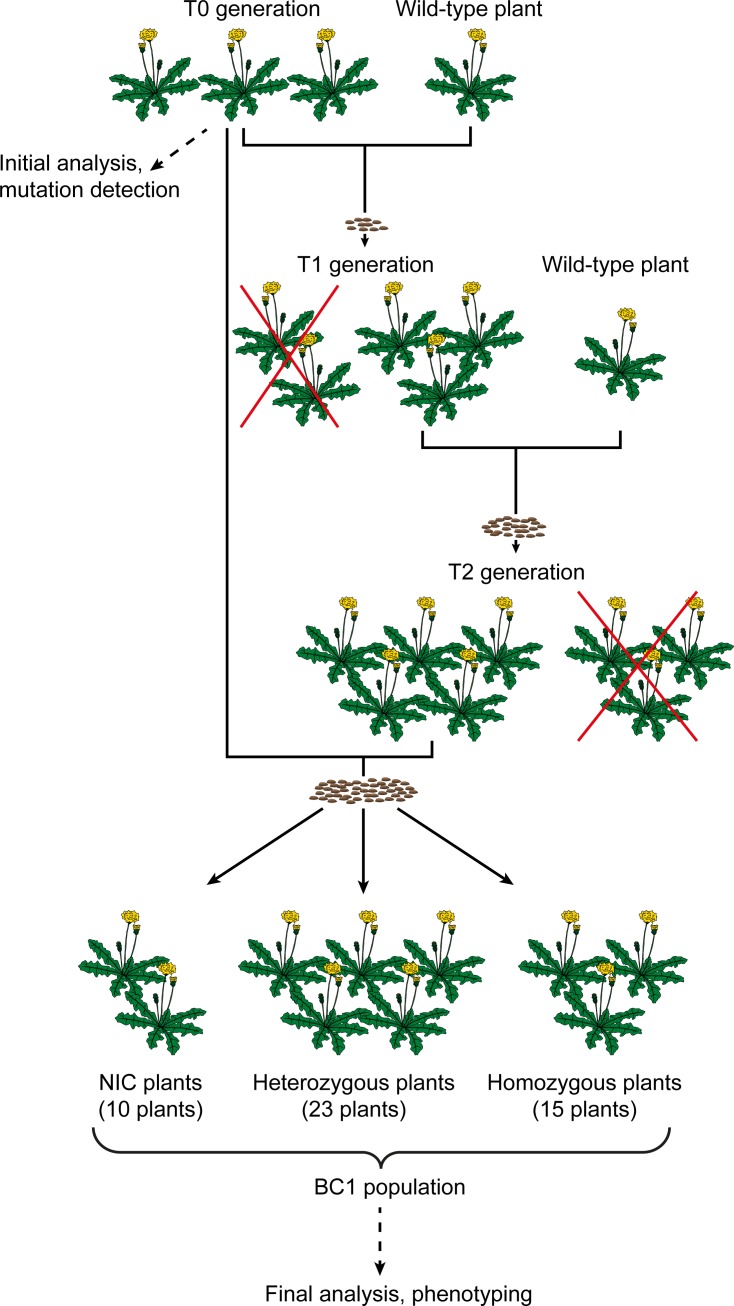
Crossing scheme for the generation of the backcross population. T0 plants were generated by stable *Agrobacterium*-mediated transformation, genome editing was analysed and plants were subsequently pollinated with a compatible *T*. *koksaghyz* wild-type plant, resulting in T1 plant families. T1 plants carrying desired mutations (not segregated out) were pollinated with another compatible *T*. *koksaghyz* wild-type plant, resulting in the corresponding T2 plant families. Plants of the T2 generation carrying desired mutations (not segregated out) were pollinated with the T0 plants, resulting in BC1 offspring consisting of 10 NIC plants, 23 heterozygous and 15 homozygous knockout plants. BC1, backcross generation 1.

*T*. *koksaghyz* is a sexually reproducing, self-incompatible species, so all three T0 plants were pollinated with the same *T*. *koksaghyz* wild-type plant to generate three corresponding T1 families. To bypass the self-incompatibility and generate homozygous plants with two mutated *RALF1* alleles, we generated T2 offspring from the T1 plants carrying the desired mutations by pollination with another compatible wild-type plant. T2 plants carrying the mutation were then backcrossed with the T0 generation as a pollen donor, which was preserved in tissue culture in the meantime. The resulting back-cross populations (BC1) (Fig 5) was analysed by FLA to detect the edited genes (S1 Appendix). This led to the identification of 10 near isogenic control (NIC) plants, 23 heterozygous knockout plants and 15 homozygous knockout plants, as anticipated due to Mendelian segregation. Seeds from the BC1 populations were sown and the plants were grown under greenhouse conditions. Twelve weeks after germination, the plants were harvested to investigate root growth and morphology. In addition to FLA, total protein extracts of harvested NIC and homozygous knockout plants of the BC1 were analysed by mass spectrometry. In homozygous knockout plants, a shorter peptide sequence was identified compared to the NIC plants (Table 1), obviously as a result of the mutation in the *TkRALFL1* gene.

**Table 1:** Analysis of crude protein extracts from NIC and homozygous knockout plants of the BC1 generation by mass spectrometry using full-length TkRALFL1 peptide sequence as reference.

	Peptide sequence
NIC	MAISTNLLLLHLLLISALIFRPAAAGTADFNWLSKQGGGCS GSIAECMVGGELEMESESTRR**ILATTNHISYGALQPNNVPCSQRGTSYYN** **CRSGGQANPYQR**GCSTITRCRR
Homozygous plants	MAISTNLLLLHLLLISALIFRPAAAGTADFNWLSKQGGGCSG SIAECMVGGELEMESESTRR**ILATTNHISYGALQPNNVPCSQRGTSYYN** **CR**SGGQANPYQRGCSTITRCRR

Peptides identified by mass spectrometry are highlighted by bold black letters.

**Fig 6:**
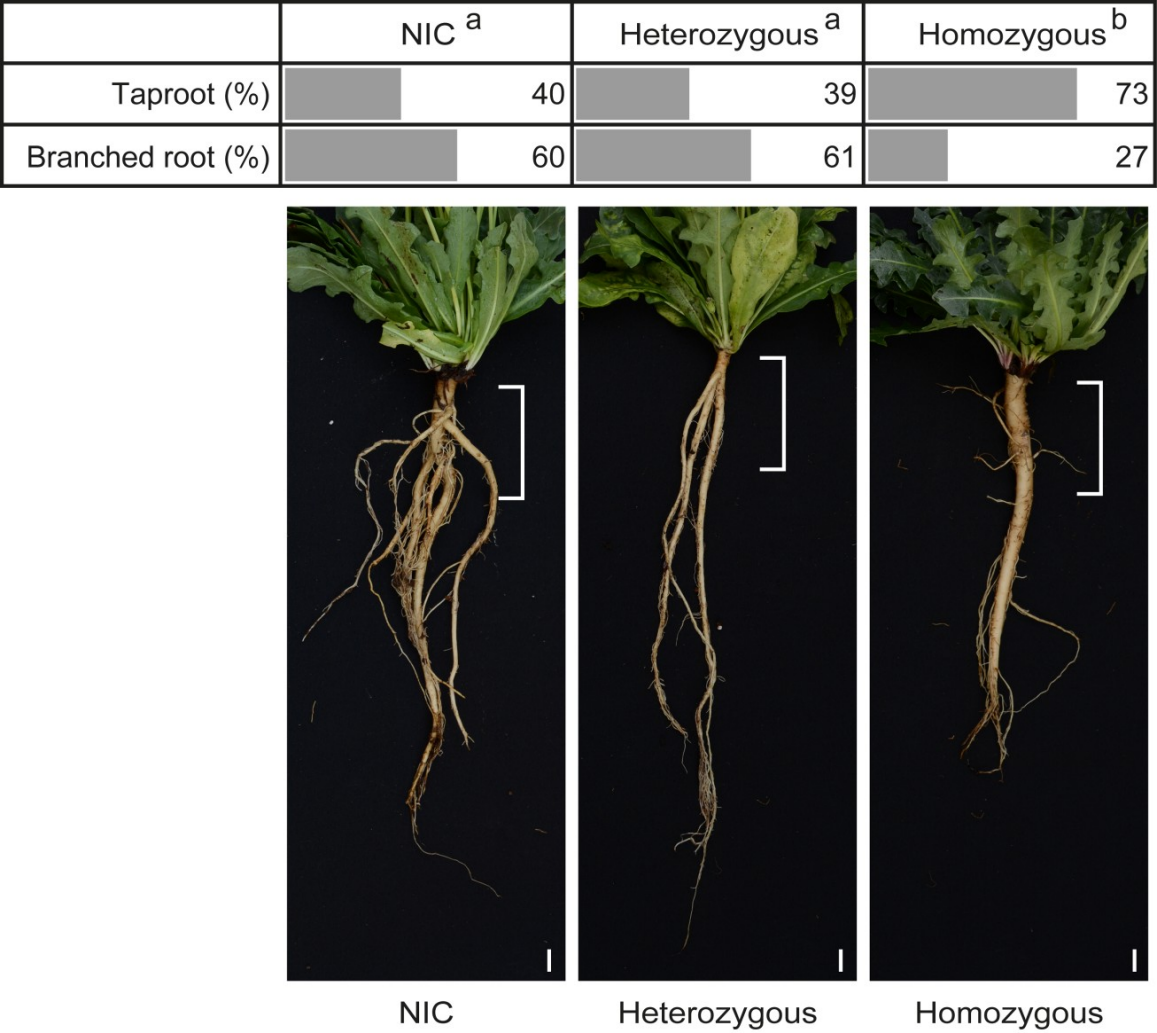
Effect of *TkRALFL1* knockout by CRISPR/Cas9 on primary root phenotype of adult *T*. *koksaghyz* plants. Evaluation of the root phenotype of 12-week-old plants from the BC1 generation of the *TkRALFL1* CRISPR/Cas9 experiment; n = 10 for NIC plants, n = 23 for heterozygous plants, n = 15 for homozygous plants. Length of the bar depicts the percentage occurrence of the taproot phenotype in the individual plant categories. The root system of representative plants in each of the three categories are shown below. The white bracket at the top end of the root indicates the 5 cm section that was evaluated to determine the root phenotype, and for measuring the primary root volume described in Table 1. Pearson’s chi-square test was used to evaluate differences in root phenotype frequency between two plant groups, and differences are depicted by different letters, with p < 0.1; Scale bar = 1 cm.

All plants developed in a comparable manner above ground, but during harvest we observed a greater frequency of taproots rather than branched roots among the homozygous knockout plants (73% taproot formation). Among the heterozygous knockout plants and NIC plants, we observed 39% and 40% taproot formation, respectively. In the remaining plants, a normal branched root system was observed (Fig 6). The relationship between *TkRALFL1* knockout and the taproot phenotype was the first indication that the knockout affects the entire morphology of the developing primary root.

Next, we determined the volume of the upper 5 cm of the primary root by measuring the root diameter directly below and 5 cm below the leaf rosette. We restricted the evaluation of the root volume to the upper 5cm to avoid distorting influences on the measurement such as enhanced root branching. Furthermore, a high volume in the upper root part is of interest for breeding of crops growing below ground regarding biomass and yield, but also for simplified harvesting, comparable to chicory. The volume was ~35% higher on average in the heterozygous knockout plants and ~60% higher on average in homozygous knockout plants (Table 2). Analysis of the root dry weight (DW) revealed average increases of 48% and 35% in the heterozygous and homozygous knockout plants, respectively, compared to the NIC (Table 2). However, there was no substantial difference in the primary root length at this developmental stage (Table 2). The higher primary root volume and root dry weight appeared to correlate with the taproot morphology, with an increasing percentage of taproot formation yielding a corresponding higher root dry weight.

**Table 2:** Root characteristics of *TkRALFL1*-knockout plants.

	NIC		Heterozygous plants		Homozygous plants	
Root dry weight (g)	1.89	(±0.87)	2.80	(±1.47)	2.56	(±1.03)
Primary root volume, upper 5 cm (cm^3^)	6511	(±2930)	8773	(±4135)	10425	(±5401)
Primary root diameter below leaf rosette (mm)	7.60	(±1.50)	9.17	(±3.01)	9.38	(±2.21)
Primary root diameter five cm below leaf rosette (mm)	4.55	(±1.86)	5.70	(±1.79)	6.15	(±2.13)
Primary root length (cm)	22.03	(±8.26)	20.84	(±3.39)	19.81	(±4.22)

n = 10 for NIC plants, n = 23 for heterozygous plants, n = 15 for homozygous plants. Values are means ± SD.

### *TkRALFL1* knockout also increases the yields of inulin and natural rubber

Finally, we measured the quantity of inulin and natural rubber in the roots of nine randomly chosen plants in the homozygous knockout, heterozygous knockout and NIC categories. The inulin content was determined by enzymatic hydrolysis in aqueous root extracts followed by HPLC analysis. The heterozygous and homozygous knockout plants showed a slight increase in the inulin content compared to NIC plants (Table 3), with a similar degree of polymerization (DP) as well as similar fructose and sucrose levels (S3 Table). The natural rubber content was analysed by measuring the poly(*cis*-1,4-isoprene) level by proton nuclear magnetic resonance (^1^H-NMR) spectroscopy, showing that heterozygous and homozygous knockout plants contained slightly lower levels of natural rubber than the NIC plants (Table 3).

**Table 3:** Inulin and natural rubber content of *TkRALFL1* knockout plants.

	NIC		Heterozygous plants		Homozygous plants	
Inulin content (mg/g DW)	499	(±61)	535	(±50)	568	(±33) **
Natural rubber content (mg/g DW)	25.5	(±9.4)	21.8	(±6.0)	19.6	(±7.3)

n = 8–9; DW, dry weight; values are mean ± SD; statistical significance proved by two-tailed *t*-test,

**p<0.01.

These values were then used to calculate the inulin and natural rubber yields per plant by offsetting against the root dry weight of the respective plants. The inulin yield per heterozygous plant increased by ~44% on average compared to the NIC plants, whereas in the homozygous plants the increase was ~58% on average. The natural rubber yield in the heterozygous plants increased by ~20% and in the homozygous plants by ~14% compared to the NIC plants (Table 4).

**Table 4:** Inulin and natural rubber yield per plant of *TkRALFL1* knockout plants.

	NIC	Heterozygous plants			Homozygous plants				
Inulin yield (g/plant)	1.08	(±0.48)		1.55	(±0.81)		1.70	(±0.44) **	
Natural rubber yield (mg/plant)	50.96	(±20.05)		60.98	(±33.41)		57.93	(±22.46)	

8–9; DW, values are mean ± SD; statistical significance proved by two-tailed t-test,

**p<0.01.

## Discussion

*T*. *koksaghyz* accumulates considerable quantities of valuable metabolites such as inulin and natural rubber in its roots, and is therefore considered as a promising alternative source of both feedstocks [6,11,36]. However, most previous studies have focused on the inulin and natural rubber metabolic pathways [37–41], rather than the root morphology. In this study, for the first time, we addressed the morphology, growth and productivity of *T*. *koksaghyz* roots by investigating the impact of TkRALFL1, a member of the RALFL peptide family.

We identified 10 RALF-like sequences by *in silico* analysis of *T*. *koksaghyz* nucleotide data and based on the spatial expression pattern of each gene we identified TkRALFL1 as the most promising candidate to influence root morphology (Fig 1). This candidate showed a high percentage similarity to the AtRALF1 peptide sequence and likewise was strongly expressed in the roots on a relative expression scale [21]. Two other genes encoding peptides with high levels of similarity to AtRALF1 (TkRALFL5 and TkRALFL6) were strongly expressed in the latex compared to all other tissues including roots. Therefore, both emerged as interesting candidates that may be adressed in future studies to determine their influence on e.g. laticifer growth.

The functionality of the TkRALFL1 peptide was confirmed in a root growth assay. The exposure of young *T*. *koksaghyz* wild-type seedlings to the exogenous peptide significantly reduced primary root growth compared to controls (Fig 3). Similar results have been reported for *A*. *thaliana* [21,24,26] and tomato [26] seedlings. RALF peptides lacking the YISY motif are commonly used in the literature as control peptide [28,26]. We analysed the TkRALFL1 sequence regarding specific protease cleavage sites and by pepsin digestion at a pH of 1.3 we obtained a mature peptide lacking the first 10 amino acids including the YISY motif. The treatment of *T*. *koksaghyz* seedlings with TkRALFL1(11–49) did not affect the primary root growth compared to untreated control (S2 Fig). In accordance with the literature, this again emphasizes the importance of the YISY motif for the activity of RALF peptides [28].

Interestingly, lower concentrations of TkRALFL1 caused a stronger inhibition of root growth, which hints for a dose-dependent hormetic response. Hormetic effects have already been described for plant hormones such as gibberellin [42], or synthetic plant hormone derivatives such as 2,4-D [43].

Bergonci *et al*. (2014) showed that the reduced root growth in transgenic *A*. *thaliana* seedlings overexpressing *AtRALF1* reflected a smaller cell size. Conversely, when *AtRALF1* expression was suppressed, the root cell size increased, as did the primary root length [24]. The examination of *T*. *koksaghyz* wild-type seedlings treated with TkRALFL1 revealed a similar effect as observed for *A*. *thaliana* seedlings. All concentrations tested resulted a strongly reduced cell length, again in a hormetic dose-dependent manner. Our results fit the current model proposing that RALF1 negatively regulates cell expansion, presumably by a combination of Ca^2+^ signalling, higher MAP kinase activity, and pH modulation in the apoplast, which triggers a signalling cascade that inhibits cell elongation and growth [25–27,29]. Additionally, a hormetic effect of TkRALFL1 in *T*. *koksaghyz* could be assumed, but needs to be analysed in detail in further studies.

Based on the *AtRALF1* overexpression and silencing data in *A*. *thaliana*, we anticipated that *T*. *koksaghyz* plants lacking for TkRALFL1 would show enhanced root growth. We generated heterozygous and homozygous *TkRALFL1* T0 knockout plants using CRISPR/Cas9. Since *T*. *koksaghyz* is a self-incompatible species, we established back-cross populations consisting of NIC, heterozygous and homozygous plants (Fig 5). Although this increases the genetic background of the individual plants, the heterozygous and homozygous *TkRALFL1* knockout plants showed a higher primary root volume and root dry weight than NIC plants (Table 2). The roots of the adult plants we examined were rather thicker than longer than the NIC roots (Table 2), whereas in seedlings the knockout affected root length (Fig 3) as previously reported in *A*. *thaliana* seedlings [24]. More importantly, we also observed a difference in the overall root morphology. Homozygous knockout plants more frequently developed a single taproot than heterozygous knockout plants or NIC plants, which more frequently developed a branched root system (Fig 6). In *A*. *thaliana* seedlings, the knockdown of *AtRALF1* conversely yielded a higher density of lateral roots [24], similar to the knockdown of *AtRALFL34* [44].

TkRALFL1 therefore appears to fulfil a role similar to AtRALF1 and may involve related signalling pathways in each species causing enhanced root growth after knockout, but the precise effects may differ between developmental stages. AtRALF1, the best-studied plant RALF peptide, is known to influence the phosphorylation state of the receptor kinase FERONIA and other proteins [21]. When AtRALF1 binds to FERONIA, it recruits the receptor-like kinase RIPK [45] which modulates the intracellular Ca^2+^ level [29,46]. Furthermore, FERONIA inhibits proton transport by the H^+^-ATPase AHA2, thus changing the pH of the apoplast and inhibiting cell elongation and growth [21]. BRI1-associated receptor kinase 1 (BAK1) is another AtRALF1 interaction partner [47]. BAK1 is involved in several signalling pathways related to growth and development, but mainly participates in the response to brassinosteroid hormones, which are important regulators of plant cell growth and development [48]. Dressano *et al*. (2017) proposed a role for BAK1 in the activation of AtRALF1-inducible genes, which mediate cell wall rearrangement and thus affect cell expansion and growth [24,49]. AtRALF1 is also involved in stomatal closure [50] and furthermore, recently published studies addressing different receptor-like kinases and RALF peptides in receptor-ligand interactions and affinities indicate that there is still little known about the exact molecular mechanisms of RALF peptides and subsequent signalling cascades [51–53]. Taken together, these studies indicate that many different signalling pathways involved in root growth and development can be affected by RALF peptides and may therefore be modulated by TkRALFL1 in *T*. *koksaghyz* to produce the observed taproot phenotype in the knockout plants. In order to elucidate, whether the observed morphological changes are caused by downstream cascades affected by TkRALFL1, further studies are necessary. In particular, these might address auxin- and brassinosteroid-dependent regulations, since both plant hormones are involved in plant root growth. As long as potential effects on downstream cascades remain unclarified, it is likely that the final root growth is influenced by various factors. However, we identified a clear effect of TkRALFL1 on the early root development. The identification and characterization of *T*. *koksaghyz* orthologues of AtRALFL34, whose sequence and phenotype was published by Murphy *et al*. (2016) while the present study was already in progress, might provide further information about root phenotype development in *T*. *koksaghyz* and thus might be addressed in future studies. And moreover, the root architecture among dicot species is diverse, so the molecular mechanisms underlying the effect of TkRALFL peptides on taproot formation and lateral root development need to be investigated in different species.

The *TkRALFL1* knockout plants not only showed differences in root morphology, but also changes in the inulin and natural rubber content (Table 3). The inulin levels were higher in the knockouts than in NIC plants, whereas natural rubber levels were lower. Inulin is stored in parenchymal root cells near to the natural rubber-containing laticifers, which form concentric rings within the roots [54]. The increased inulin content may reflect the enlarged root cells that develop in the absence of *TkRALFL1*, which potentially have a greater capacity for inulin storage. The slight decrease in the natural rubber content may reflect the tendency towards taproot formation and the higher primary root volume in the knockout plants. Javorsky (1944) reported that the size of laticifers does not necessarily correlate with the root size, and that smaller roots may contain more laticifers per unit area than larger roots. Plants with taproots and a higher primary root volume may therefore produce less natural rubber per unit dry weight than plants with a branched root system, where natural rubber can additionally be contained in the lateral roots. However, the lower natural rubber content per unit dry weight in the *TkRALFL1*-knockout plants was more than compensated by the higher overall root dry weight, such that the total natural rubber yield per plant was higher in the knockout plants than the NIC plants (Table 4). This same phenomenon also affected the inulin yield, with the rather small increase in inulin content per unit dry weight converted into a much larger increase in yield per plant (Table 4). Given that the total rubber yield was even higher in the heterozygous knockout plants than the homozygous ones, a TkRALFL1 dosage effect may influence the rubber content and this should be taken into account when developing an optimal rubber-producing root phenotype. Expression data of *TkRALFL1* obtained from *T*. *koksaghyz* cultivars with variable inulin and natural rubber yields might be useful to approach that, as well as correlation studies concerning *TkRALFL1* expression in cultivars with variable root phenotypes and biomasses.

The improvement in the total yields for both inulin and natural rubber is necessary to enhance the productivity of *T*. *koksaghyz* and thus its value as an alternative source of these feedstocks. Taken together, the total carbon storage of the knockout plants increased substantially. Previous studies have revealed a crossover between the natural rubber and inulin metabolic pathways such that the energy from inulin degradation can be redirected to natural rubber biosynthesis [41]. This should be taken into account for future combinatorial approaches aiming to develop profitable *T*. *koksaghyz* genotypes for commercial cultivation. The taproot phenotype of the *TkRALFL1*-knockout plants substantially improves the agronomic performance of *T*. *koksaghyz* as a potential industrial crop because single taproots are much easier to harvest than branched roots. They can be pulled from the soil easily and the wasted yield of lateral roots left in the soil is minimized.

A taproot phenotype also allows a higher planting density, increasing the total biomass (and subsequently the total natural rubber and inulin yield) per unit of cultivated land area [11,55]. In field trials, Kreuzberger *et al*. (2016) doubled the biomass, rubber and inulin yields when the density of dandelion plants per hectare was almost quadrupled and the inter-row distance was halved. Harvesting the roots of dense-growing plants could be facilitated by favouring the development of single taproots using modern breeding approaches. If the root phenotype generated by the *TkRALFL1* knockout can be replicated in future experiments using standard mutagenesis approaches and field trials under standard cultivation conditions, then the *TkRALFL1* locus can be used as a marker to select for varieties with improved agronomic performance.

## Materials and methods

### Plant material and cultivation

*T*. *koksaghyz* plants (wild-type and genome-edited lines) were grown in the greenhouse at 14°C-18°C (night) and 22°C-25°C (day) and 20 klx light intensity (high pressure sodium lamp, HPS 600 Watts, Greenbud, enhanced yellow and red spectrum) with a 16-h photoperiod. The plants were cultivated in a pre-fertilized mixture (1:1) of standard soil (ED73 Einheitserde, Fröndenberg, Germany) and garden mold (Botanical Garden Münster, Germany), and were fertilized every 4 weeks with a commercial fertilizer according to the manufacturer’s recommendations (Hakaphos Plus, Compo GmbH, Münster, Germany). For phenotyping of genome-edited lines, the roots were harvested after 12 weeks and measured in length with a ruler and in diameter with a digital calliper. The root system phenotype and primary root volume was evaluated using the uppermost 5 cm of the root to exclude root branching caused by peat residues or soil compaction. The roots were then quick-frozen, lyophilized and ground to powder prior to further analysis. *T*. *koksaghyz* seeds for the root growth assay were generated by pollinating a single wild-type mother plant with nine other wild-type plants, and the seeds were harvested only from the mother plant. This reduced the genetic variation among the progeny plants, given that *T*. *koksaghyz* is a sexually reproducing species which undergoes obligatory outcrossing.

### Extraction of genomic DNA and total RNA, and cDNA synthesis

Genomic DNA was extracted from *T*. *koksaghyz* leaf tissue using the NucleoSpin Plant II Kit (Macherey-Nagel, Düren, Germany) according to the manufacturer’s instructions. Prior to RNA extraction, latex was harvested by cutting the stem with a razor blade and harvesting the expelling latex in 90 μl rubber extraction buffer (REB) with 5 mM DTT as previously described [56]. Roots, leaves and flowers were lyophilized and ground to powder. Total RNA was extracted from all *T*. *koksaghyz* tissues using the innuPREP Plant RNA Kit (Analytik Jena, Jena, Germany) according to the manufacturer’s instructions. cDNA was synthesized using PrimeScript Reverse Transcriptase Master Mix (TAKARA, Clontech, Sain-Germain-en-Laye, France) according to the manufacturer’s instructions.

### Amplification of full-length *TkRALFL* sequences

The coding sequences of *TkRALFL1–TkRALFL10* were amplified from *T*. *koksaghyz* root cDNA and the genomic sequences of were amplified from *T*. *koksaghyz* genomic DNA using gene-specific oligonucleotides (S4 Table). Each cDNA sequence and the corresponding genomic DNA sequence were aligned using SeqMan software (DNASTAR Inc., Madison, Wisconsin, USA) to identify the positions of potential introns. N-terminal signal peptides were predicted using SignalP (http://www.cbs.dtu.dk/services/SignalP/).

### Gene expression analysis by quantitative real-time PCR

Quantitative real-time PCR was carried out as previously described [39] with slight modifications. Samples for spatial expression analysis represented 6–9 technical replicates of 2–3 cDNA pools consisting of cDNA from four individual plants each, diluted 1:30. The reference genes elongation factor 1α (*Tkef1α*) and ribosomal protein L27 (*TkRP*) were used for the normalization of gene expression [40] and expression levels were calculated using Bio-Rad CFX Manager v3.1 software (Bio-Rad Laboratories Inc., Hercules, California, USA). Oligonucleotides used for qPCR analysis and the corresponding primer efficiencies are listed in S4 and S5 Tables, respectively.

### Heterologous *TkRALFL1* expression in *E*. *coli*

The *TkRALFL1* mature peptide sequence was amplified from *T*. *koksaghyz* genomic DNA using the oligonucleotides proTkRALFL1-NcoI-fwd and proTkRALFL1-XhoI-rev (S4 Table), and introduced into the Gateway pENTR4 vector at the NcoI and XhoI restriction sites. The *TkRALFL1* mature peptide sequence was then transferred to the Gateway pDEST17 vector by Gateway LR recombination, yielding expression vector pDEST17+TkRALFL1 with an N-terminal His-tag. The integrity of the final construct was confirmed by sequencing using the oligonucleotide T7_promoter (S4 Table). For the empty vector control, the *ccdB* cassette was removed from pDEST17 by digesting with NotI and SalI followed by religation. The constructs were introduced into *E*. *coli* BL21 cells for recombinant protein expression according to the manufacturer’s instructions (Merck, Darmstadt, Germany). Soluble and insoluble protein fractions from the cells were separated and visualized by SDS-PAGE and western blot analysis. For protein purification, the pellet was quick-frozen and disrupted by sonication before purification on Ni-NTA agarose (Qiagen, Hilden, Germany) according to the manufacturer’s instructions with slight modifications. The protein was eluted using additional elution buffer F (100 mM NaH_2_PO_4_, 10 mM Tris, 8 M urea, pH 3.3), buffer G (100 mM NaH_2_PO_4_, 10 mM Tris, 8 M urea, pH 2.2) and buffer G+ (100 mM NaH_2_PO_4_, 10 mM Tris, 8 M urea, 500 mM NaCl, pH 3.3). The protein was dialyzed against 0.1% (v/v) formic acid, lyophilized and dissolved in 0.1% (v/v) formic acid prior to visualization by SDS-PAGE and subsequent use in the root growth assay.

### Pepsin digestion of heterologous expressed TkRALFL1

After purification, the solution containing the heterologous expressed TkRALFL1 peptide dissolved in 0.1% (v/v) formic acid was further acidified with 1N HCl until a pH of 1.3. Pepsin (Roth, Karlsruhe, Germany) was added in a final concentration of 0.4% (w/v) and the solution was incubated at 37°C for 4 hours. For purification of the resulting peptides, a 30 kDa Amicon Ultra-4 Centrifugal Filter Unit (Merck Millipore, Burlington, Massachusetts, USA) was used in the first step to exclude the remaining pepsin (4,000 g, 20 min; three washing steps with 4 ml 0.1% (v/v) formic acid each). The flow-through was collected and loaded on a 3kDa Amicon Ultra-4 Centrifugal Filter Unit. After three washing steps with 4 ml 0.1% (v/v) formic acid each (4,000 g, 30 min), the remaining supernatant was collected and analysed by SDS-PAGE prior to use in the root growth assay.

### SDS-PAGE and western blot analysis

His-TkRALFL1 is a small protein, so we used a tricine-containing SDS-PAGE system [57]. The protein was separated on two consecutive polyacrylamide gels containing 10% and 16% acrylamide/bisacrylamide, respectively, and transferred to a PVDF membrane. The gel was stained with Coomassie Brilliant Blue or PageBlue Protein Staining solution as indicated. For western blot analysis, the membrane was incubated with a mouse IgG specific for the His-tag conjugated to horseradish peroxidase (Sigma-Aldrich, St. Louis, Missouri, USA) diluted 1:5000 in PBS + 5% (w/v) skimmed milk powder for 1 h. After washing, the His-tagged protein was visualized using SuperSignal West Dura Extended Duration Substrate (Thermo Fisher Scientific, Waltham, Massachusetts, USA).

### Root growth assay and cell dimension measurement

*T*. *koksaghyz* seeds were surface sterilized in 70% ethanol for 2 min and in 6% sodium chlorate plus 0.1% Triton X-100 for 7 min, followed by three washes in sterile water. The seeds were plated on Hoagland medium plates (1.6 g/L Hoagland’s No. 2 Basal Salt Mixture (Sigma), 1.8% (w/v) Low Melting Agarose (Duchefa), pH 5.8) and cold treated (4°C) for 24 h, then germinated vertically at 26°C with a 16-h photoperiod (20 klux). After 7 days, the seedlings were transferred to new Hoagland medium plates containing His-TkRALFL1 in different concentrations or a non-peptide control. His-TkRALFL1 was dissolved in 0.1% (v/v) formic acid to prevent protein precipitation. Non-peptide control plates contained the same volume of 0.1% (v/v) formic acid as the other plates. The pH of all plates was adjusted with 15 mM MES-KOH (pH 6.5) to maintain the medium at pH 5.8. After transfer, the position of the root tips was marked on the plate and the change in root length was determined after 5 and 10 days using ImageJ software (https://imagej.nih.gov/ij/download.html). For determination of the cell length, root tips were stained with propidium iodide (0.1 mM) and images were acquired using confocal laser scanning microscopy (CLSM) with excitation and emission wavelength of 555 nm and 566–718 nm, respectively. The most inner 6 rows of rhizodermal cells were measured in a distance of 1 mm from the very tip and upwards using ImageJ software.

### Cloning of CRISPR/Cas9 construct and plant transformation procedures

The expression vector system compatible with Gateway cloning (Thermo Fisher Scientific) was obtained from Holger Puchta (Karlsruhe Institute of Technology, Karlsruhe, Germany) and was used as previously described [58] with slight modifications. The native resistance cassette was replaced with a phosphinothricin resistance cassette from vector pFGC5941, consisting of the mannopine synthase promoter, phosphinothricin resistance gene, and mannopine synthase polyA signal [59]. The protospacer specific for *TkRALFL1* was designed using the ATUM design tool (https://www.atum.bio/eCommerce/cas9/input) and two oligonucleotides with the corresponding sequences (S4 Table) were aligned to prepare the protospacer. The integrity of the final construct was confirmed by sequencing using the oligonucleotide CRISPR-Seq (S4 Table). *T*. *koksaghyz* plants were transformed as previously described [38] with slight modifications. After transformation, *T*. *koksaghyz* leaf discs were incubated on callus induction medium containing 400 mg/L amoxicillin. Shoot growth was promoted on shoot induction medium supplemented with 1 mg/L kinetin, 100 mg/L indole acetic acid and 200 mg/L amoxicillin, and for root growth the shoots were transferred to root induction medium supplemented with 400 mg/L amoxicillin.

### DNA fragment length analysis

DNA fragments were amplified from genomic DNA using forward primer 6FAM-TkRALFL1_fwd, which is labelled with the blue fluorescent dye 6FAM, and reverse primer TkRALFL1_rev or TkRALFL1_downstream_rev (S4 Table). PCR products were premixed with the LIZ600 marker (Applied Biosystems, Waltham, Massachusetts, USA) and analysed using the ABI 3730 Genetic Analyzer (Applied Biosystems) according to the manufacturer’s instructions. Results were evaluated using GeneMarker v2.6.4 software (SoftGenetics, State College, Pennsylvania, USA).

### Characterization of TkRALFL1 peptide by mass spectrometry

His-TkRALFL1 produced in E. coli was isolated by SDS-PAGE and the corresponding band was cut from the gel. Protein analysis via mass spectrometry was performed as previously described [41]. For characterization of TkRALFL1 peptide in BC1 NIC and transgenic plants, powdered root material was boiled in reducing protein loading buffer and separated by SDS-PAGE. A slice of the gel encompassing approx. 3–15 kDA was cut out and washed three times with Millipore water for 30 s. The protein bands were subjected to trypsin digestion overnight as previously described [60]. Next, the peptides were resuspended in 100 μl 0.5% acetic acid and desalted using C18 Empore Stage Tips. The samples were loaded and centrifuged (4,000 rpm, 1.5 min), reloaded 3 times, then washed with 200 μl 0.5% acetic acid and centrifuged (4000 rpm, 3 min). Next, the peptides were eluted in 200 μl 60% acetonitrile containing 0.5% acetic acid and centrifuged (4,000 rpm, 2 min), and two times eluted in 200 μl 80% acetonitrile containing 0.5% acetic acid and centrifuged (4,000 rpm, 2 min). The solution was dried by vacuum centrifugation and resuspended in 12 μl 5% acetonitrile containing 0.5% trifluoroacetic acid for analysis by mass spectrometry. The chromatographic separation of peptides was performed using an Ultimate 3000 RSLCnano System (Dionex, Thermo Fisher Scientific, Darmstadt, Germany). The mobile phase for the loading pump was 0.05% (v/v) ultrapure water (A) and 80% acetonitrile / 0.05% (v/v) trifluoroacetic acid in ultrapure water (B). 2μl of the sample was loaded on a trapping column (C18 PepMap 100, 300 μM x 5 mm, 5 μm particle size, 100 Å pore size; Thermo Scientific) and desalted for 5 min using eluent A at a flow rate of 10 μl/min. The trap column was switched online with the separation column (Acclaim PepMap100 C18, 75 μm x 50 cm, 2 μM particle size, 100 Å pore size (Thermo Fisher Scientific)). The mobile phases for elution of the peptides from the column were 0.1% (v/v) formic acid in ultrapure water (A*) and 80% acetonitrile / 0.1% (v/v) formic acid in ultrapure water (B*). The peptides were eluted at a flow rate of 250 nl min-1 using the following gradient profile: 2.5–18% B* over 65 min, 18–32% B* over 50 min, 32–99% B* over 5 min, 99% B* over 20 min. The column was re-equilibrated with 97.5% A* for 30 min. The LC system was coupled via a nanospray source to a Q Exactive Plus mass spectrometer (Thermo Finnigan, Thermo Fisher Scientific). Full scans (m/z 375–1600) were acquired in positive ion mode by FT-MS in the Orbitrap at a resolution of 70,000 (FWHM) with internal lock mass calibration on m/z 445.12003. An inclusion list with the precursor masses of the in silico digested proteins of interest was created using the FT Programs from Thermo Scientific version 2.0.70702. The 12 most intense ions were fragmented with 27% normalized collision energy at a resolution of 17,500 and maximal IT of 120 ms. Automatic gain control (AGC) was enabled with target values of 3 x 106 and 5 x 104 for MS full scans and MS/MS, respectively. One microscan was acquired per MS/MS spectrum and maximum ion trap fill time was 50 ms [61]. Dynamic exclusion was enabled with an exclusion duration of 30s, a repeat count of 1 and exclusion mass width of ± 5 ppm. Unassigned charge states, single charged and charge ≥ 5 were rejected. Peptide identification against a database comprising the target peptide sequence as well as the reversed peptide sequence as decoy was performed as previously described [41]. Raw data (mgf-file) of mass spectrometry analyses are included in the supporting information.

### Determination of poly(*cis*-1,4-isoprene) by ^1^H-NMR spectroscopy

Poly(*cis*-1,4-isoprene) levels in *T*. *koksaghyz* root material were determined by ^1^H-NMR spectroscopy as previously described [41]. In brief, 100 mg of ground root material was supplemented with 1500 μl of a mixture with 10% toluene-d8, tetramethylsilane and 16 mM 2,6-dimethoxyphenol (DMOP) as internal standards. After extraction at 20°C with shaking at 1000 rpm for 16 h, the samples were centrifuged at 21,000 g for 110 min. The supernatant was then analysed using a Bruker Avance III 400 MHz spectrometer with a 5-mm broadband inverse (BBI) probe head (Bruker, Billerica, Massachusetts, USA) at 298 K. Data were acquired using a one-dimensional ^1^H-NMR pulse program with 90° pulse and a relaxation delay of 20 s. The C5 methyl signal for poly(*cis*-1,4-isoprene) was integrated at 1.75 ppm and the methyl signal of DMOP at 3.34 ppm for quantitative analysis.

### Determination of inulin levels by HPLC

Inulin, sucrose and fructose levels were determined by HPLC as previously described [41]. In brief, approx. 100 mg ground root material was supplemented with the tenfold volume of HPLC-grade water and boiled for 18 h at 85°C, after that centrifuged at 5,000 g for 20 min. 125 μl of the supernatant was mixed with 125 μl acetate buffer (20 mM, pH 4.15), another 125 μl of the supernatant was mixed with 122.5 μl acetate buffer and 2.5 μl (110 U/l) *Aspergillus niger* inulinase (Sigma-Aldrich). Both reactions were incubated for 2 h at 55°C and 700 rpm, then stopped by adding 1 mM EDTA (pH 8.0), followed by centrifugation for 2 min at 13,000 g. Chicory inulin was used as control. For determination of inulin content, the fructose, glucose and sucrose levels of undigested and digested samples were determined by HPLC using RID-10A refractive index detector (Shimadzu, Duisburg, Germany) and the Asahipak NH2P-50 4E column (Shodex, Mainz, Germany). Acetonitrile with HPLC-grade water (75:25 v/v) was used as the mobile phase with a flow rate of 1 ml/min. Calculation of the inulin content and degree of polymerization (DP) was done as described by [41].

### Statistical analysis

All statistical analyses were performed using OriginPro 2019 (OriginLab, Northampton, Massachusetts, USA). The Kolmogorov-Smirnov test was used to check for normal distribution of gene expression levels, root growth, cell length and metabolic data. Statistical significance of gene expression levels, root growth, cell length and metabolic data was proven by two-tailed *t*-test. Pearson’s chi-square test was used to evaluate differences in root phenotype frequency.

### Accession numbers

The GenBank identifiers for genes used in this study are TkRALFL1 (MG872314), TkRALFL2 (MG872315), TkRALFL3 (MG872316), TkRALFL4 (MG872317), TkRALFL5 (MG872318), TkRALFL6 (MG872319), TkRALFL7 (MG872320), TkRALFL8 (MG872321), TkRALFL9 (MG872322), TkRALFL10 (MG872323).

## Supporting information

S1 Fig Spatial expression profiles of *TkRALFL1–TkRALFL10* in 12-week-old wild-type *T*. *koksaghyz* plants. Normalized expression in flower, leaf, root and latex tissue was determined by qPCR. The mRNA levels were normalized using the reference genes *Tkef1α* and *TkRP*. Values are means ± standard deviation; n = 8–12. Statistical significant differences were proven by two-tailed *t*-test and are depicted by asterisks with *p<0.05, **p<0.01 and ***p<0.001.
(TIF)

S2 Fig Effect of TkRALFL1(11–49) on primary root growth. (A) Mature TkRALFL1 sequence with cleavage site for pepsin at a pH of 1.3 (indicated by black arrow) as predicted by ExPASy PeptideCutter analysis tool. (B) SDS-PAGE analysis of a non-digested control as well as purified recombinant His-TkRALFL1 after pepsin digestion (equals TkRALFL1(11–49)). (C) Increase of the primary root length of *T*. *koksaghyz* wild-type seedlings after treatment for 5 days with 0.1 μM TkRALFL1(11–49) peptide dissolved in buffer (0.1% (v/v) formic acid). Values were normalized to the mean growth increase of seedlings treated with pure buffer was as non-peptide control. n = 26–27, normal distribution proven by Kolmogorov-Smirnov test, two-tailed *t*-test showed no statistical significant difference, box plot as described in Fig 2.
(TIF)

S3 Fig DNA fragment length analysis and sequencing results of *TkRALFL1* T0 knockout plants. (A) The electropherogram of the wild-type plant shows one predominant peak depicting a DNA fragment length of 339 nt. The electropherogram of the first transgenic plant shows one peak shifted by +1 nt, and one peak shifted by –2 nt. The other two transgenic plants show one peak shifted by +1 nt and one peak with the wild-type size. DNA fragments were amplified using primers 6FAM-TkRALFL1_fwd and TkRALFL1_rev. (B) The sequencing of the transgenic knockout plants revealed different mutations compared to the wild-type sequence, which is shown above the electropherograms.
(TIF)

S4 Fig Investigation of off-targets in CRISPR/Cas9 plants. (A) Nucleotide alignment of the *TkRALFL1* protospacer and *TkRALFL1–TkRALFL10* sequences using the BLAST algorithm. Query = protospacer specific for *TkRALFL1*. The PAM sequence is underlined in black, and the protospacer seed region is underlined in orange. *TkRALFL4*, *TkRALFL5* and *TkRALFL7* show no similarity. (B) Sequencing results of *TkRALFL6* sequence after amplification from the wild-type and transgenic plants used in this study. Sequence of the *TkRALFL1* protospacer is depicted below the electropherograms with the PAM sequence underlined in black and seed region underlined in orange. The mismatches within the seed region are highlighted by the black boxes.
(TIF)

S1 Table Distance matrix of the MUSCLE alignment shown in Fig 1A. The numbers represent the distances calculated for each pair of sequences, converted to percent identity. Percent identities of AtRALF1 compared to TkRALFL1, TkRALFL5 and TkRALFL6 are highlighted with orange boxes.
(DOCX)

S2 Table Analysis of TkRALFL1 produced in *E*.*coli* by mass spectrometry. The specific peptides covered 61.64% of the expressed His-TkRALFL1 as target sequence.
(DOCX)

S3 Table Degree of polymerization of inulin, and fructose and sucrose levels in *TkRALFL1*-knockout plants. Values are means ± SD (n = 8–9). DP, degree of polymerization; FM, fructose molecules; DW, dry weight.
(DOCX)

S4 Table List of oligonucleotides used in this study. Sequences are shown in 5’→3’ direction.
(DOCX)

S5 Table Oligonucleotide efficiencies for qPCR. All efficiencies were determined by melt curve analysis at 66°C.
(DOCX)

S1 Appendix DNA fragment length analysis of *TkRALFL1*-knockout plants, back-cross 1 (BC1) population. The electropherogram of the wild-type control plant showed one predominant peak depicting a DNA fragment length of 379 nt, taken as a reference to evaluate the electropherograms of the transgenic BC1 plants. Electropherograms of the BC1 plants are pre-sorted by their evaluation as near isogenic control, heterozygous, or homozygous plants. Each DNA fragment was amplified using the primers 6FAM-TkRALFL1_fwd and TkRALFL1_downstream_rev.
(PDF)

S2 Appendix Raw data (mgf-file) of mass spectrometry analysis of recombinant His-TkRALFL1 peptide. (MGF)

S3 Appendix Raw data (mgf-file) of TkRALFL1 peptide identification by mass spectrometry analysis in BC1 NIC plant. (MGF)

S4 Appendix Raw data (mgf-file) of TkRALFL1 peptide identification by mass spectrometry analysis in BC1 transgenic homozygous plant. (MGF)
