# Supplementary figures and images for: Loss of function mutation of the Rapid Alkalinization Factor (RALF1)-like peptide in the dandelion Taraxacum koksaghyz entails a high-biomass taproot phenotype
Source: PLoS One. 2019 May 24;14(5):e0217454. doi: 10.1371/journal.pone.0217454 (PMC6534333; doi:10.1371/journal.pone.0217454)

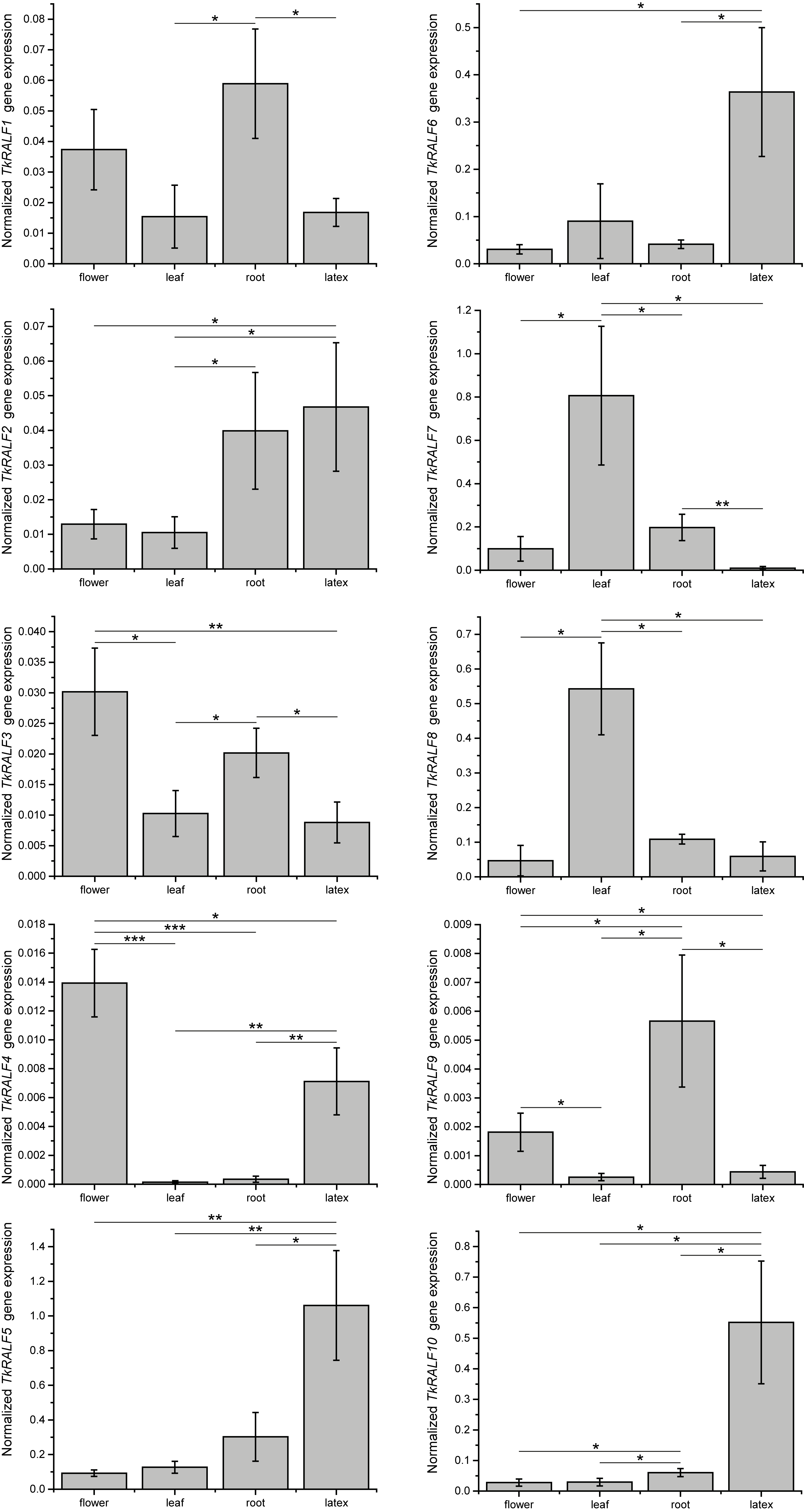

Supplement: S1 Fig — Normalized expression in flower, leaf, root and latex tissue was determined by qPCR. The mRNA levels were normalized using the reference genes Tkef1α and TkRP. Values are means ± standard deviation; n = 8–12. Statistical significant differences were proven by two-tailed t-test and are depicted by asterisks with *p<0.05, **p<0.01 and ***p<0.001. (TIF) [file pone.0217454.s001.tif]

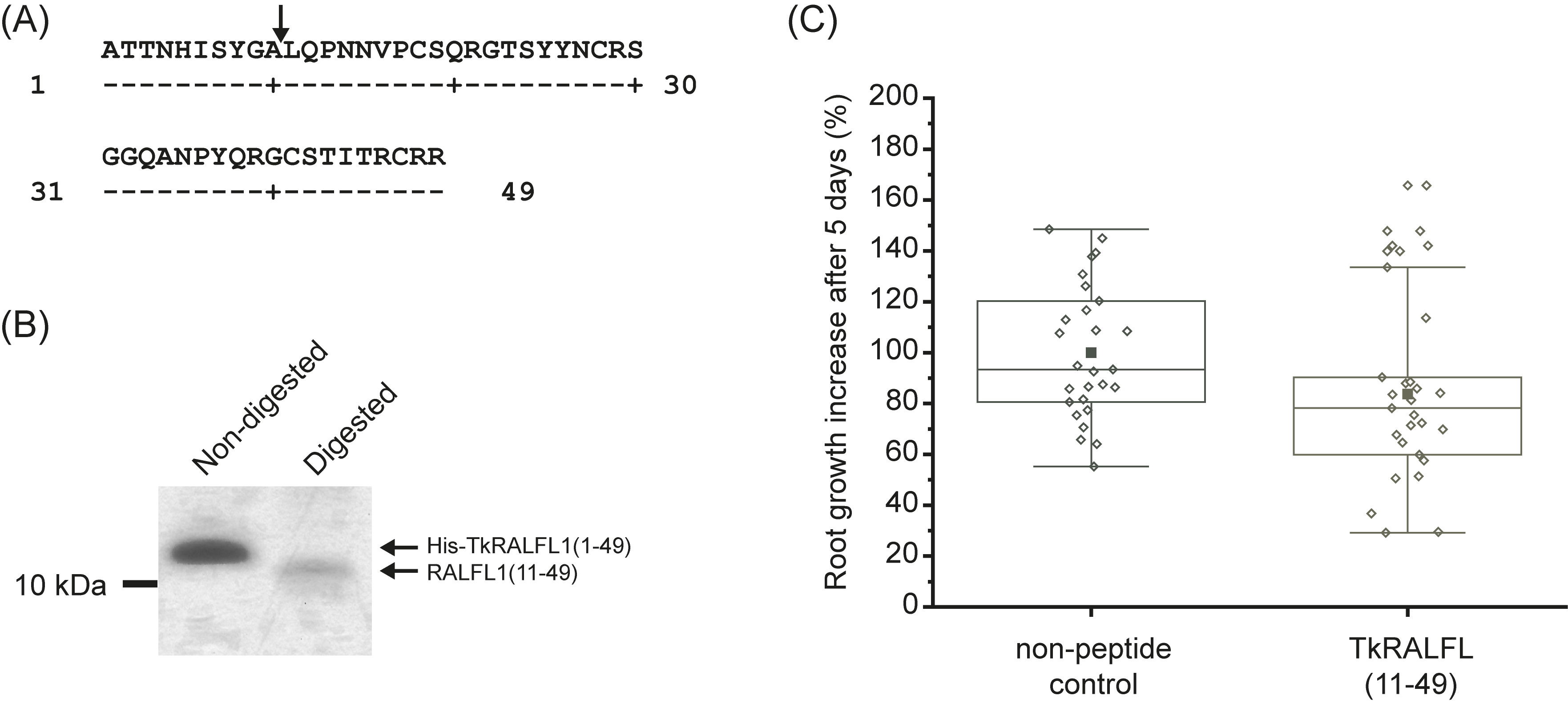

Supplement: S2 Fig — (A) Mature TkRALFL1 sequence with cleavage site for pepsin at a pH of 1.3 (indicated by black arrow) as predicted by ExPASy PeptideCutter analysis tool. (B) SDS-PAGE analysis of a non-digested control as well as purified recombinant His-TkRALFL1 after pepsin digestion (equals TkRALFL1(11–49)). (C) Increase of the primary root length of T. koksaghyz wild-type seedlings after treatment for 5 days with 0.1 μM TkRALFL1(11–49) peptide dissolved in buffer (0.1% (v/v) formic acid). Values were normalized to the mean growth increase of seedlings treated with pure buffer was as non-peptide control. n = 26–27, normal distribution proven by Kolmogorov-Smirnov test, two-tailed t-test showed no statistical significant difference, box plot as described in Fig 2. (TIF) [file pone.0217454.s002.tif]

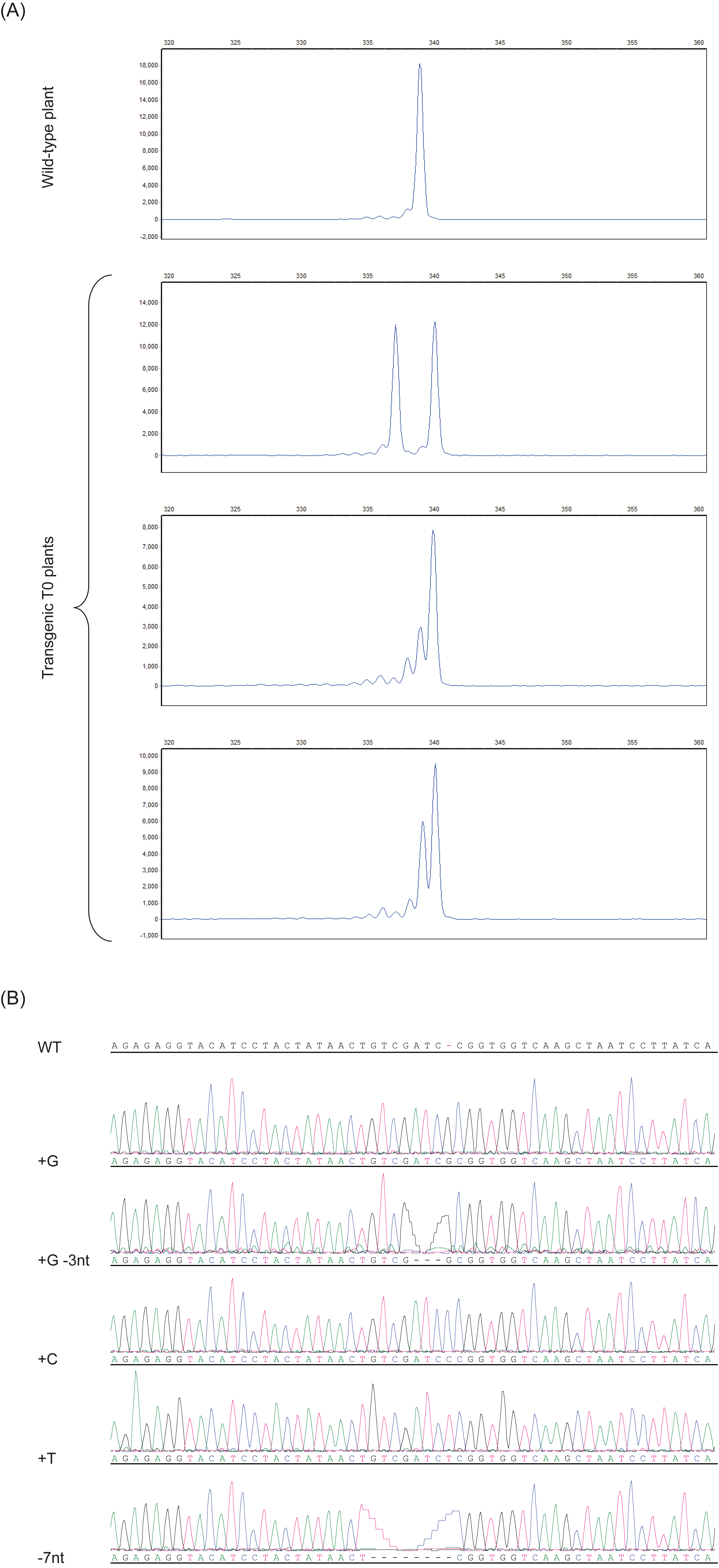

Supplement: S3 Fig — (A) The electropherogram of the wild-type plant shows one predominant peak depicting a DNA fragment length of 339 nt. The electropherogram of the first transgenic plant shows one peak shifted by +1 nt, and one peak shifted by –2 nt. The other two transgenic plants show one peak shifted by +1 nt and one peak with the wild-type size. DNA fragments were amplified using primers 6FAM-TkRALFL1_fwd and TkRALFL1_rev. (B) The sequencing of the transgenic knockout plants revealed different mutations compared to the wild-type sequence, which is shown above the electropherograms. (TIF) [file pone.0217454.s003.tif]

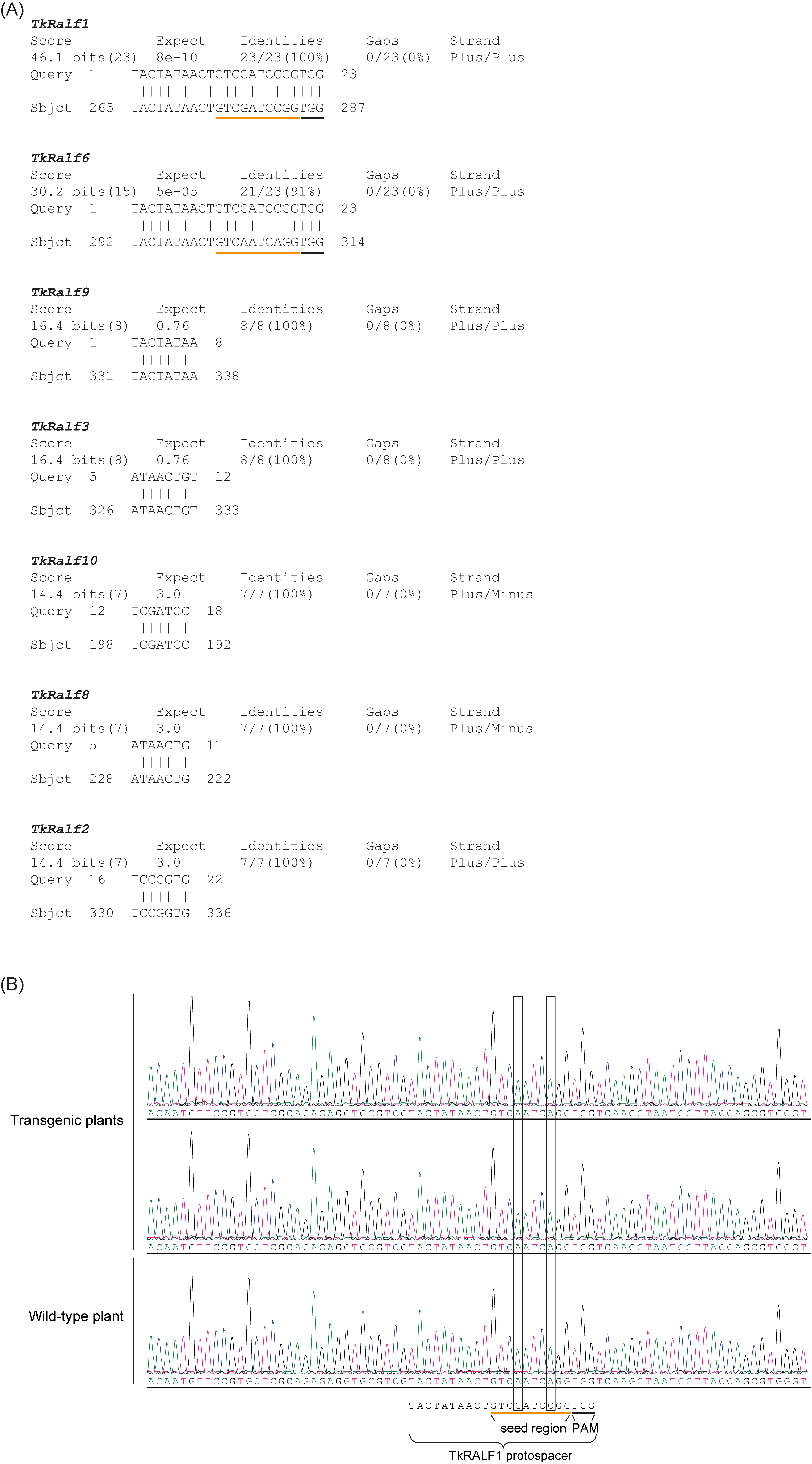

Supplement: S4 Fig — (A) Nucleotide alignment of the TkRALFL1 protospacer and TkRALFL1–TkRALFL10 sequences using the BLAST algorithm. Query = protospacer specific for TkRALFL1. The PAM sequence is underlined in black, and the protospacer seed region is underlined in orange. TkRALFL4, TkRALFL5 and TkRALFL7 show no similarity. (B) Sequencing results of TkRALFL6 sequence after amplification from the wild-type and transgenic plants used in this study. Sequence of the TkRALFL1 protospacer is depicted below the electropherograms with the PAM sequence underlined in black and seed region underlined in orange. The mismatches within the seed region are highlighted by the black boxes. (TIF) [file pone.0217454.s004.tif]
