# Supplementary material for: Loss of function mutation of the Rapid Alkalinization Factor (RALF1)-like peptide in the dandelion Taraxacum koksaghyz entails a high-biomass taproot phenotype
Source: PLoS One. 2019 May 24;14(5):e0217454. doi: 10.1371/journal.pone.0217454 (PMC6534333; doi:10.1371/journal.pone.0217454)
Supplement: S1 Table — The numbers represent the distances calculated for each pair of sequences, converted to percent identity. Percent identities of AtRALF1 compared to TkRALFL1, TkRALFL5 and TkRALFL6 are highlighted with orange boxes. (DOCX) [file pone.0217454.s005.docx]

**S1 Table. Distance matrix of the MUSCLE alignment shown in Fig 1A.** The numbers represent the distances calculated for each pair of sequences, converted to percent identity. Percent identities of AtRALF1 compared to TkRALFL1, TkRALFL5 and TkRALFL6 are highlighted with orange boxes.

|  | TkRALFL1 | TkRALFL2 | TkRALFL3 | TkRALFL4 | TkRALFL5 | TkRALFL6 | TkRALFL7 | TkRALFL8 | TkRALFL9 | TkRALFL10 |
| --- | --- | --- | --- | --- | --- | --- | --- | --- | --- | --- |
| AtRALF1 | 62 | 43 | 39 | 46 | 62 | 62 | 43 | 39 | 41 | 57 |
| TkRALFL1 |  | 46 | 48 | 56 | 59 | 85 | 41 | 43 | 48 | 54 |
| TkRALFL2 |  |  | 64 | 44 | 51 | 43 | 30 | 28 | 33 | 41 |
| TkRALFL3 |  |  |  | 44 | 46 | 46 | 28 | 30 | 34 | 44 |
| TkRALFL4 |  |  |  |  | 49 | 56 | 33 | 28 | 38 | 44 |
| TkRALFL5 |  |  |  |  |  | 59 | 39 | 39 | 41 | 52 |
| TkRALFL6 |  |  |  |  |  |  | 39 | 41 | 43 | 51 |
| TkRALFL7 |  |  |  |  |  |  |  | 36 | 30 | 33 |
| TkRALFL8 |  |  |  |  |  |  |  |  | 28 | 36 |
| TkRALFL9 |  |  |  |  |  |  |  |  |  | 44 |
