# Supplementary material for: Loss of function mutation of the Rapid Alkalinization Factor (RALF1)-like peptide in the dandelion Taraxacum koksaghyz entails a high-biomass taproot phenotype
Source: PLoS One. 2019 May 24;14(5):e0217454. doi: 10.1371/journal.pone.0217454 (PMC6534333; doi:10.1371/journal.pone.0217454)
Supplement: S2 Table — The specific peptides covered 61.64% of the expressed His-TkRALFL1 as target sequence. (DOCX) [file pone.0217454.s006.docx]

**S2 Table. Analysis of TkRALFL1 produced in *E.coli* by mass spectrometry.** The specific peptides covered 61.64% of the protein using the full-length TkRALFL1 as target sequence.

| Peptides |
| --- |
| KAGSTMATTNHISYGALQPNNVPCSQR |
| ALQPNNVPCSQR |
| ISYGALQPNNVPCSQRGTSYYNCR |
| SGGQANPYQR |
| AGSTMATTNHISYGALQPNNVPCSQR |
| GTSYYNCR |
