# Supplementary material for: Loss of function mutation of the Rapid Alkalinization Factor (RALF1)-like peptide in the dandelion Taraxacum koksaghyz entails a high-biomass taproot phenotype
Source: PLoS One. 2019 May 24;14(5):e0217454. doi: 10.1371/journal.pone.0217454 (PMC6534333; doi:10.1371/journal.pone.0217454)
Supplement: S3 Table — Values are means ± SD (n = 8–9). DP, degree of polymerization; FM, fructose molecules; DW, dry weight. (DOCX) [file pone.0217454.s007.docx]

**S3 Table. Degree of polymerization of inulin, and fructose and sucrose levels in *TkRALFL1*-knockout plants.**

|  | NIC plants | | Heterozygous plants | | Homozygous plants | |
| --- | --- | --- | --- | --- | --- | --- |
| Mean DP (FM ≥ 1) | 7.20 | (±0.81) | 7.75 | (±1.54) | 6.91 | (±0.59) |
| Fructose content (mg/g DW) | 31.79 | (±6.64) | 32.88 | (±8.52) | 36.41 | (±10.56) |
| Sucrose content (mg/g DW) | 26.37 | (±6.30) | 23.37 | (±9.81) | 31.38 | (±3.61) |

Values are means ± SD (n = 8–9). DP, degree of polymerization; FM, fructose molecules; DW, dry weight.
