# Supplementary material for: Loss of function mutation of the Rapid Alkalinization Factor (RALF1)-like peptide in the dandelion Taraxacum koksaghyz entails a high-biomass taproot phenotype
Source: PLoS One. 2019 May 24;14(5):e0217454. doi: 10.1371/journal.pone.0217454 (PMC6534333; doi:10.1371/journal.pone.0217454)
Supplement: S4 Table — Sequences are shown in 5’→3’ direction. (DOCX) [file pone.0217454.s008.docx]

**S4 Table. List of oligonucleotides used in this study.** Sequences are shown in 5’🡪3’ direction.

| Name | Sequence 5’🡪3’ | Usage |
| --- | --- | --- |
| TkRALFL1_fwd | ATGGCAATTTCTACTAATCTTCTT | Full-length amplification |
| TkRALFL1_rev | TCAACGCCGGCAACGAGTAATGG | Full-length amplification |
| TkRALFL2_fwd | ATGGCGTCTCCGCTTCTTCT | Full-length amplification |
| TkRALFL2_rev | TCACCGGCGGCAACGAGTGA | Full-length amplification |
| TkRALFL3_fwd | ATGGCGCCATCTCTTCTTCT | Full-length amplification |
| TkRALFL3_rev | TCACCGGCGGCAACGAGTGA | Full-length amplification |
| TkRALFL4_fwd | ATGGCTAATCATTTTGTGTC | Full-length amplification |
| TkRALFL4_rev | TCATCGGCCACCACACCGAG | Full-length amplification |
| TkRALFL5_fwd | ATGTCGAAGTTTGCTGGTCT | Full-length amplification |
| TkRALFL5_rev | TCAACGGCGGCAACGAGTGA | Full-length amplification |
| TkRALFL6_fwd | ATGGCGATTTCGACCAACTT | Full-length amplification |
| TkRALFL6_rev | TCAACGTTGACAACGAGTAA | Full-length amplification |
| TkRALFL7_fwd | ATGGCTGCAAGAAGCAGGTA | Full-length amplification |
| TkRALFL7_rev | CTAATGACCTTTATCGCAAG | Full-length amplification |
| TkRALFL8_fwd | ATGTTCAATGGGCGAAGAAC | Full-length amplification |
| TkRALFL8_rev | TCATGACCGACATCGTTCAT | Full-length amplification |
| TkRALFL9_fwd | ATGGAACCAGCGATAACCAT | Full-length amplification |
| TkRALFL9_rev | TCATCTACTACACTTATAGT | Full-length amplification |
| TkRALFL10_fwd | ATGAAATCCCTTTTTCTACT | Full-length amplification |
| TkRALFL10_rev | TTATGTGTTTATAGCATCTC | Full-length amplification |
| TkRALFL1_qPCR_fwd | TTCTTCTCCACCTGCTGCTG | qPCR analysis |
| TkRALFL1_qPCR_rev | ACAACCTCCTCCTTGCTTCG | qPCR analysis |
| TkRALFL2_qPCR_fwd | ACTCGCGCTTCAATTGCCGAC | qPCR analysis |
| TkRALFL2_qPCR_rev | AGATCTACGACCAGCAACCGCC | qPCR analysis |
| TkRALFL3_qPCR_fwd | TGTACGCGCCGACGTATCAG | qPCR analysis |
| TkRALFL3_qPCR_rev | CGGTGACGAGATGCAGCGAG | qPCR analysis |
| TkRALFL4_qPCR_fwd | CGACTCGCGAGAGACGCCAC | qPCR analysis |
| TkRALFL4_qPCR_rev | CCCGTCCACCTAGGACTCGTCG | qPCR analysis |
| TkRALFL5_qPCR_fwd | ACATGATCGCGCTCGCATTTCC | qPCR analysis |
| TkRALFL5_qPCR_rev | CACGGCATCTGCTGCCGAC | qPCR analysis |
| TkRALFL6_qPCR_fwd | TGGCGATTTCGACCAACTTGCTTCT | qPCR analysis |
| TkRALFL6_qPCR_rev | CGGCAGCCGGACTGGTGAAAG | qPCR analysis |
| TkRALFL7_qPCR_fwd | GCCAGTCCGTGCAATAGCACC | qPCR analysis |
| TkRALFL7_qPCR_rev | CGCCTATGCTCTTCGGCGTCC | qPCR analysis |
| TkRALFL8_qPCR_fwd | GGGCGAAGAACCCCAATCTGC | qPCR analysis |
| TkRALFL8_qPCR_rev | AGCCGCCGCCTCACAGTC | qPCR analysis |
| TkRALFL9_qPCR_fwd | CCACCACAACACCGCCTTCC | qPCR analysis |
| TkRALFL9_qPCR_rev | CCACCGCCGCTACATGCC | qPCR analysis |
| TkRALFL10_qPCR_fwd | CAACTGTAAGGGGAAAGGGGTCG | qPCR analysis |
| TkRALFL10_qPCR_rev | GCATCTCTCGCACATCTCGTGAT | qPCR analysis |
| ef1α_qPCR_fwd | CGAGAGATTCGAGAAGGAAGC | qPCR analysis |
| ef1α_qPCR_rev | CTGTGCAGTAGTACTTGGTGG | qPCR analysis |
| TkRP_qPCR_fwd | CGTCGATCTCAAGGATGTTGTC | qPCR analysis |
| TkRP_qPCR_rev | GGAGCTTTGAGAAGAACCAACG | qPCR analysis |
| TkRALFL1_proto_fwd | ATTGTACTATAACTGTCGATCCGG | CRISPR/Cas9 protospacer |
| TkRALFL1_proto_rev | AAACCCGGATCGACAGTTATAGTA | CRISPR/Cas9 protospacer |
| CRISPR-Seq | TCCCAGGATTAGAATGATTAGG | Sequencing of CRISPR/Cas9 vector |
| proTkRALFL1-NcoI-fwd | AAACCATGGCTACGACGAACCACATAAGT | Heterologous expression |
| proTkRALFL1-XhoI-rev | AAACTCGAGTCAACGCCGGCAACGAGTAA | Heterologous expression |
| T7_promotor | TAATACGACTCACTATAGG | Sequencing of expression vector |
| 6FAM-TkRALFL1_fwd | 6FAM-ATGGCAATTTCTACTAATCTTCTT | Amplification for FLA, 5‘-6FAM label |
| TkRALFL1_downstream_rev | AATCAAGTCAGGAGTAAACAGCA | Amplification for FLA |
| Cas9_fwd | GCTCCCTAAGCACTCTTTGCTC | *Cas9* amplification |
| Cas9_rev | CCTTCCCATCACCTTCACCAAC | *Cas9* amplification |
| GAPDH_fwd | CTTCAGAGAGATGATGTT | *GAPDH* amplification |
| GAPDH rev | CTTCCACCTCTCCAGTCCTT | *GAPDH* amplification |
