# Supplementary material for: Loss of function mutation of the Rapid Alkalinization Factor (RALF1)-like peptide in the dandelion Taraxacum koksaghyz entails a high-biomass taproot phenotype
Source: PLoS One. 2019 May 24;14(5):e0217454. doi: 10.1371/journal.pone.0217454 (PMC6534333; doi:10.1371/journal.pone.0217454)
Supplement: S5 Table — All efficiencies were determined by melt curve analysis at 66°C. (DOCX) [file pone.0217454.s009.docx]

**S5 Table. Oligonucleotide efficiencies for qPCR.** All efficiencies were determined by melt curve analysis at 66°C.

| Name of primer pair | Primer efficiency (%) |
| --- | --- |
| TkRALFL1_qPCR | 103.5 |
| TkRALFL2_qPCR | 103.6 |
| TkRALFL3_qPCR | 110.4 |
| TkRALFL4_qPCR | 98.9 |
| TkRALFL5_qPCR | 101.3 |
| TkRALFL6_qPCR | 97.2 |
| TkRALFL7_qPCR | 105.9 |
| TkRALFL8_qPCR | 101.2 |
| TkRALFL9_qPCR | 89.8 |
| TkRALFL10_qPCR | 119.7 |
| ef1α_qPCR | 100.0 |
| TkRP_qPCR | 104.8 |
