# Supplementary material for: Loss of function mutation of the Rapid Alkalinization Factor (RALF1)-like peptide in the dandelion Taraxacum koksaghyz entails a high-biomass taproot phenotype
Source: PLoS One. 2019 May 24;14(5):e0217454. doi: 10.1371/journal.pone.0217454 (PMC6534333; doi:10.1371/journal.pone.0217454)
Supplement: S1 Appendix — The electropherogram of the wild-type control plant showed one predominant peak depicting a DNA fragment length of 379 nt, taken as a reference to evaluate the electropherograms of the transgenic BC1 plants. Electropherograms of the BC1 plants are pre-sorted by their evaluation as near isogenic control, heterozygous, or homozygous plants. Each DNA fragment was amplified using the primers 6FAM-TkRALFL1_fwd and TkRALFL1_downstream_rev. (PDF) [file pone.0217454.s010.pdf]

S1 Appendix. DNA fragment length analysis of *TkRALFL1*-knockout plants, back-cross 1 (BC1) population. The electropherogram of the wild-type control plant showed one predominant peak depicting a DNA fragment length of 379 nt, taken as a reference to evaluate the electropherograms of the transgenic BC1 plants. Electropherograms of the BC1 plants are pre-sorted by their evaluation as near isogenic control, heterozygous, or homozygous plants. Each DNA fragment was amplified using the primers 6FAM-TkRALFL1_fwd and TkRALFL1_downstream_rev.

(A) Electropherogram from a wild-type *T. koksaghyz* control plant.

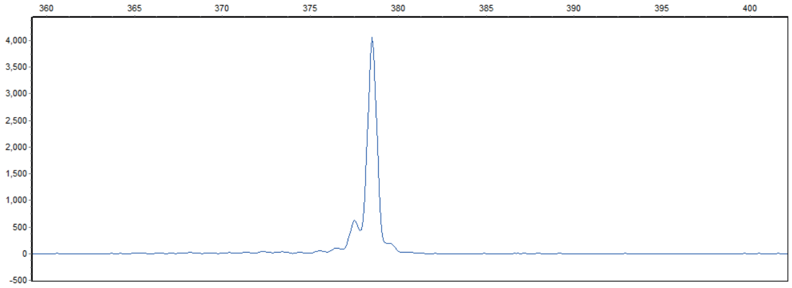

(B) Electropherograms from plants identified as near isogenic control plants.

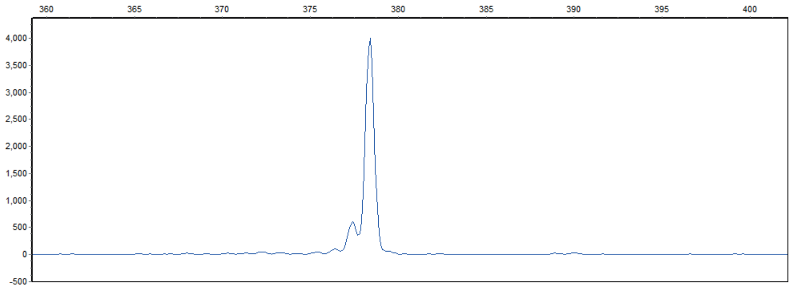

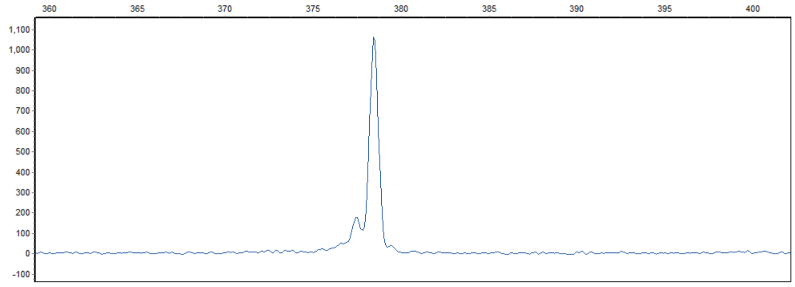

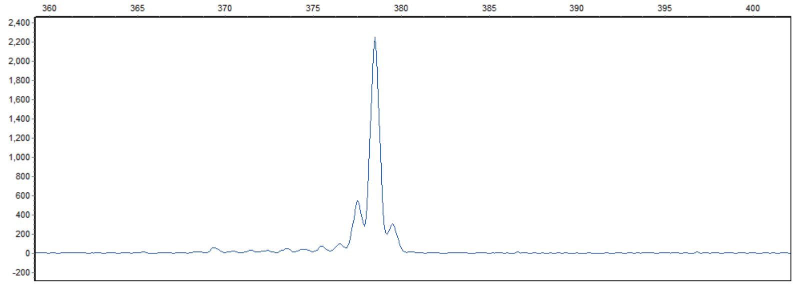

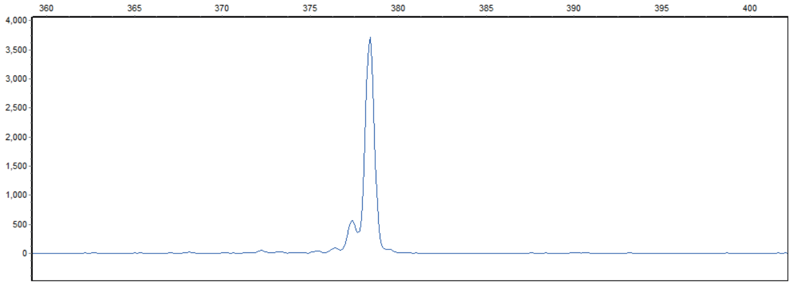

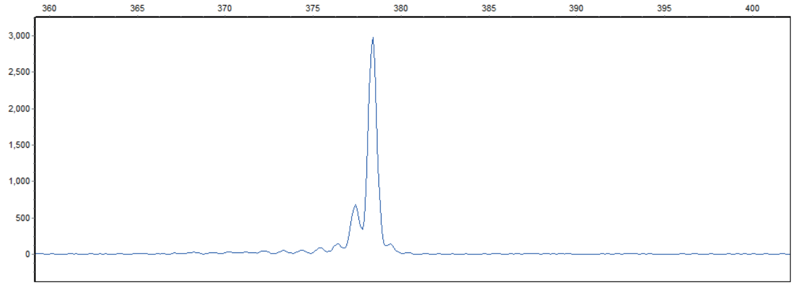
360 365 370 375 380 385 390 395 400
3,000
2,500
2,000
1,500
1,000
500
0

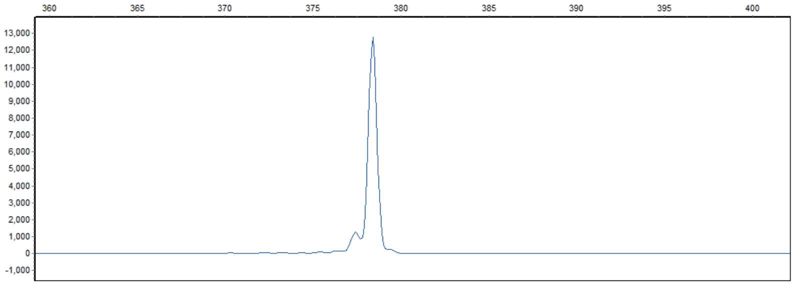
360 365 370 375 380 385 390 395 400
13,000
12,000
11,000
10,000
9,000
8,000
7,000
6,000
5,000
4,000
3,000
2,000
1,000
0
-1,000

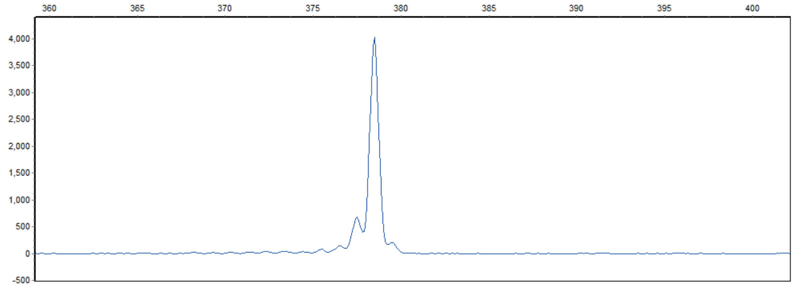
360 365 370 375 380 385 390 395 400
4,000
3,500
3,000
2,500
2,000
1,500
1,000
500
0
-500

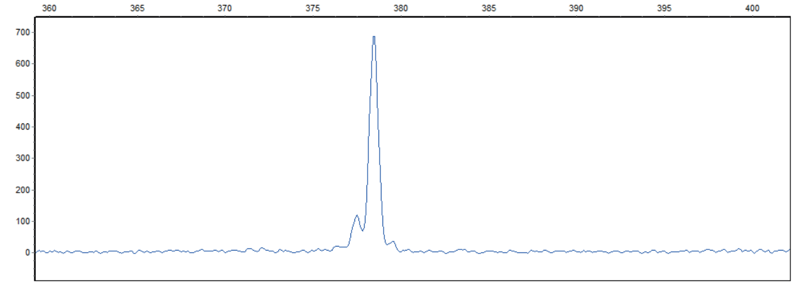
360 365 370 375 380 385 390 395 400
700
600
500
400
300
200
100
0

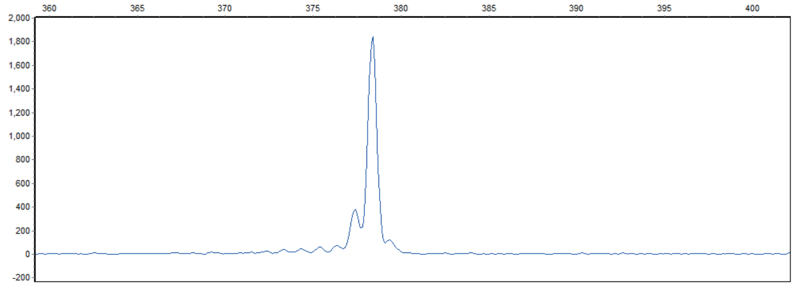
360 365 370 375 380 385 390 395 400
2,000
1,800
1,600
1,400
1,200
1,000
800
600
400
200
0
-200

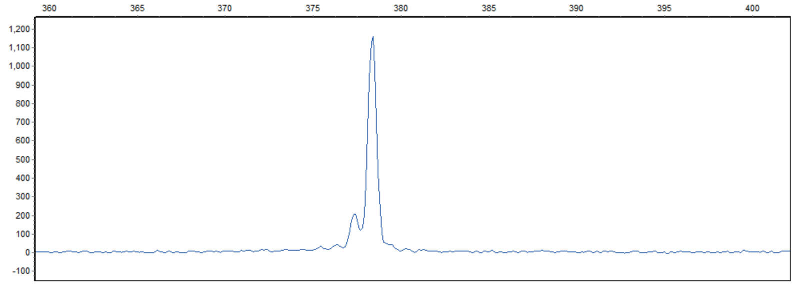
360 365 370 375 380 385 390 395 400
1,200
1,100
1,000
900
800
700
600
500
400
300
200
100
0
-100

(C) Electropherograms from plants identified as heterozygous plants.

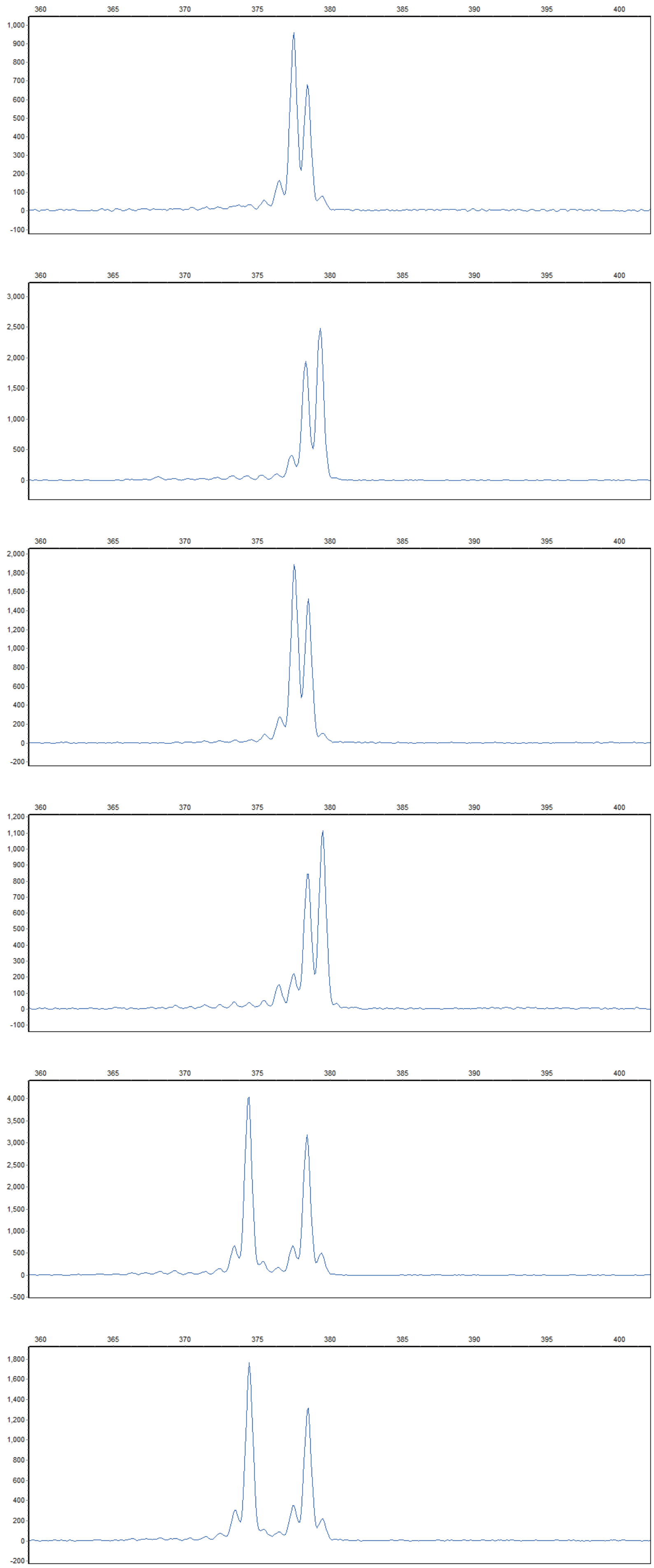

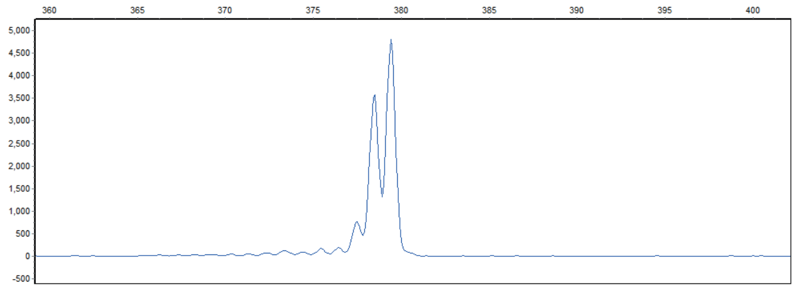
360 365 370 375 380 385 390 395 400
5,000 4,500 4,000 3,500 3,000 2,500 2,000 1,500 1,000 500 0 -500

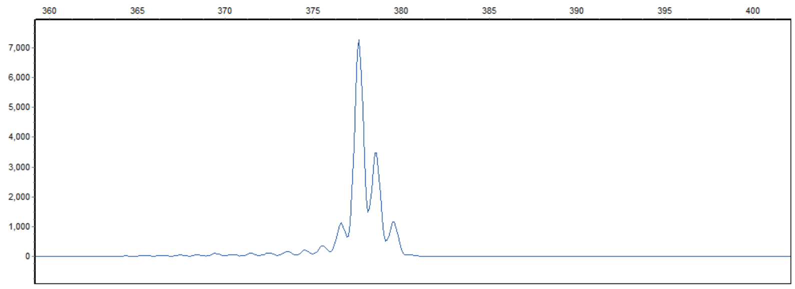
360 365 370 375 380 385 390 395 400
7,000 6,000 5,000 4,000 3,000 2,000 1,000 0

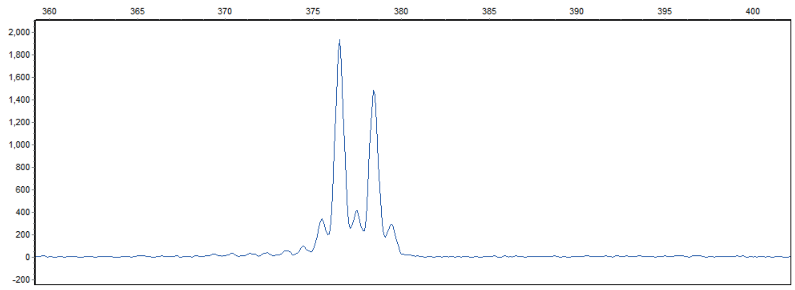
360 365 370 375 380 385 390 395 400
2,000 1,800 1,600 1,400 1,200 1,000 800 600 400 200 0 -200

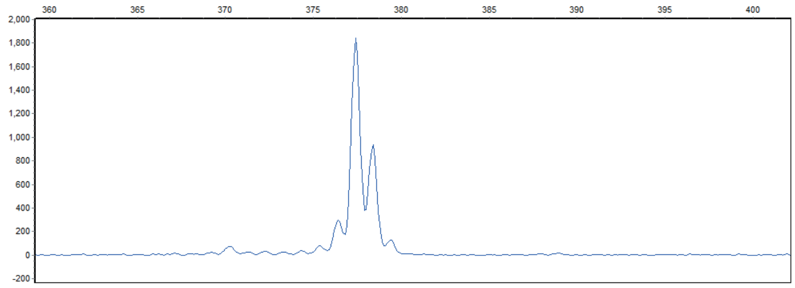
360 365 370 375 380 385 390 395 400
2,000 1,800 1,600 1,400 1,200 1,000 800 600 400 200 0 -200

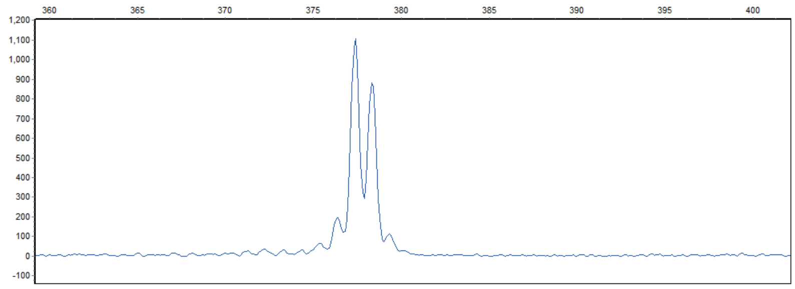
360 365 370 375 380 385 390 395 400
1,200 1,100 1,000 900 800 700 600 500 400 300 200 100 0 -100

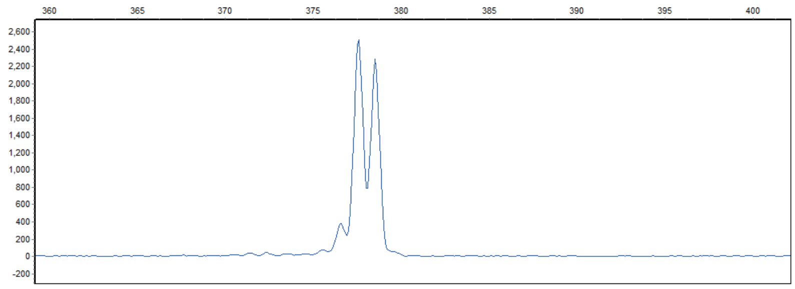
360 365 370 375 380 385 390 395 400
2,600 2,400 2,200 2,000 1,800 1,600 1,400 1,200 1,000 800 600 400 200 0 -200

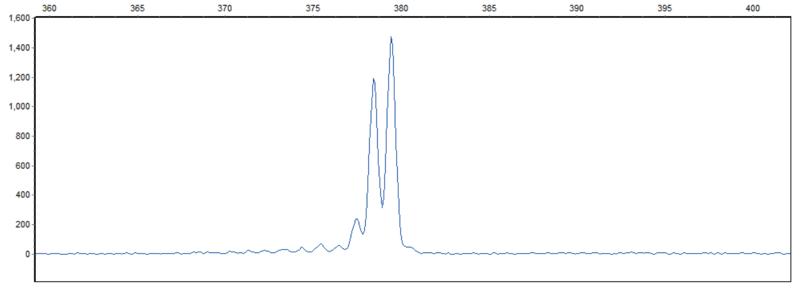
360 365 370 375 380 385 390 395 400
1,600 1,400 1,200 1,000 800 600 400 200 0

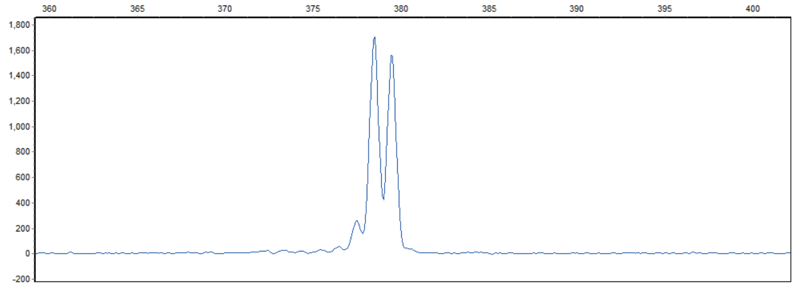
360 365 370 375 380 385 390 395 400
1,800 1,600 1,400 1,200 1,000 800 600 400 200 0 -200

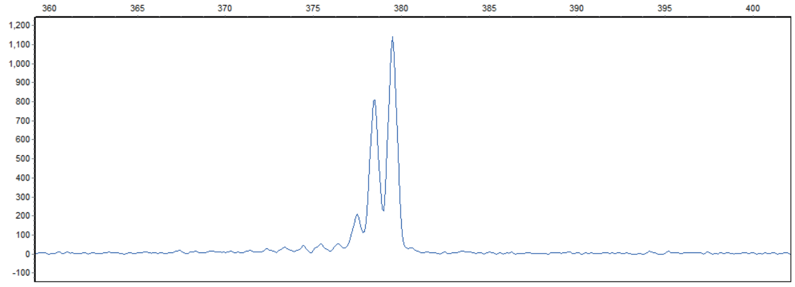
360 365 370 375 380 385 390 395 400
1,200 1,100 1,000 900 800 700 600 500 400 300 200 100 0 -100

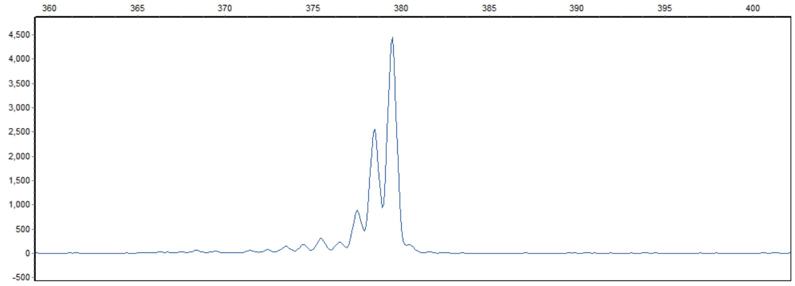
360 365 370 375 380 385 390 395 400
4,500 4,000 3,500 3,000 2,500 2,000 1,500 1,000 500 0 -500

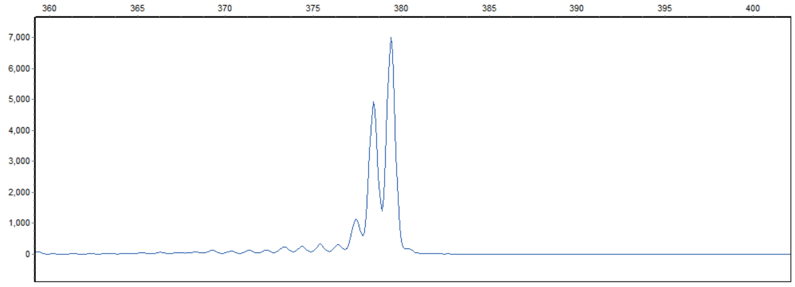
360 365 370 375 380 385 390 395 400
7,000 6,000 5,000 4,000 3,000 2,000 1,000 0

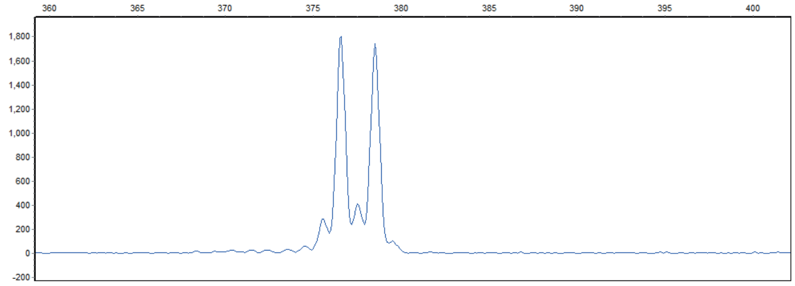
360 365 370 375 380 385 390 395 400
1,800 1,600 1,400 1,200 1,000 800 600 400 200 0 -200

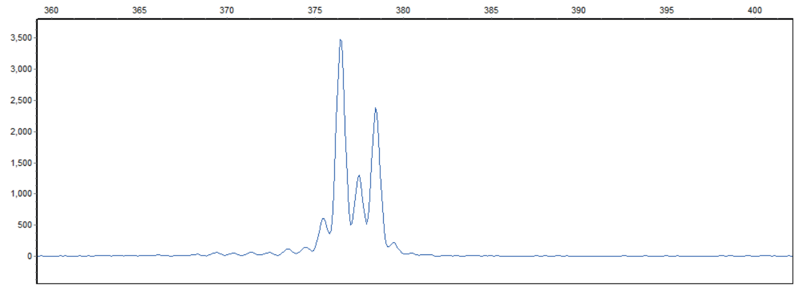
360 365 370 375 380 385 390 395 400
3,500 3,000 2,500 2,000 1,500 1,000 500 0

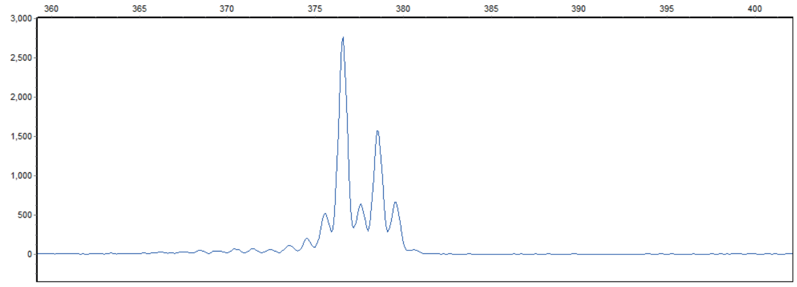
360 365 370 375 380 385 390 395 400
3,000 2,500 2,000 1,500 1,000 500 0

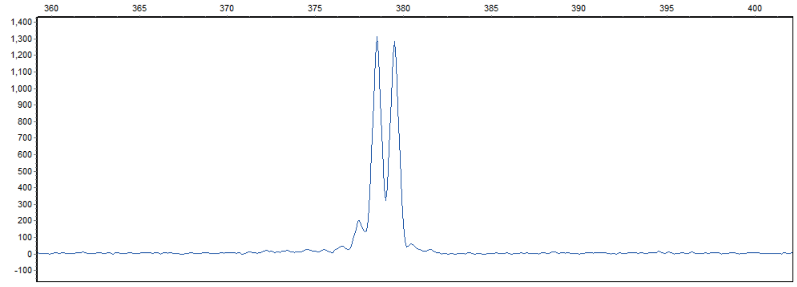
360 365 370 375 380 385 390 395 400
1,400 1,300 1,200 1,100 1,000 900 800 700 600 500 400 300 200 100 0 -100

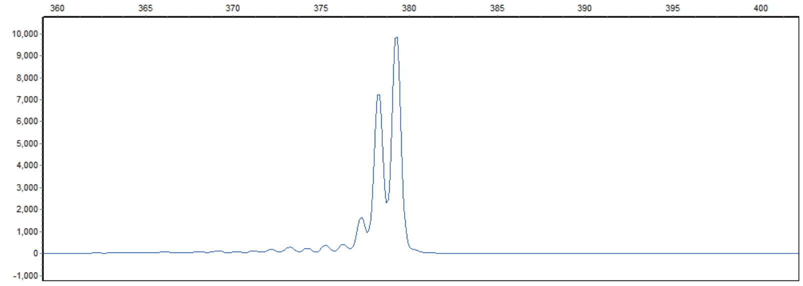
360 365 370 375 380 385 390 395 400
10,000 9,000 8,000 7,000 6,000 5,000 4,000 3,000 2,000 1,000 0 -1,000

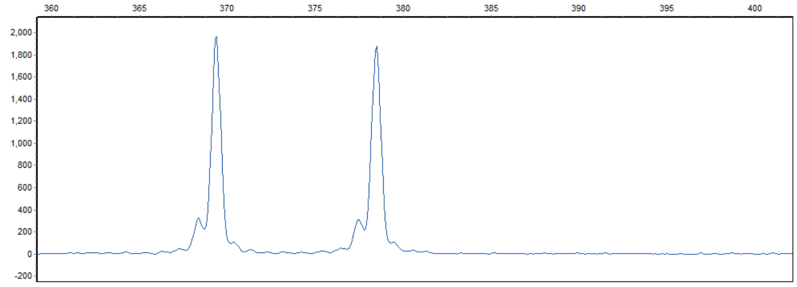
360 365 370 375 380 385 390 395 400
2,000 1,800 1,600 1,400 1,200 1,000 800 600 400 200 0 -200

(D) Electropherograms from plants identified as homozygous plants.

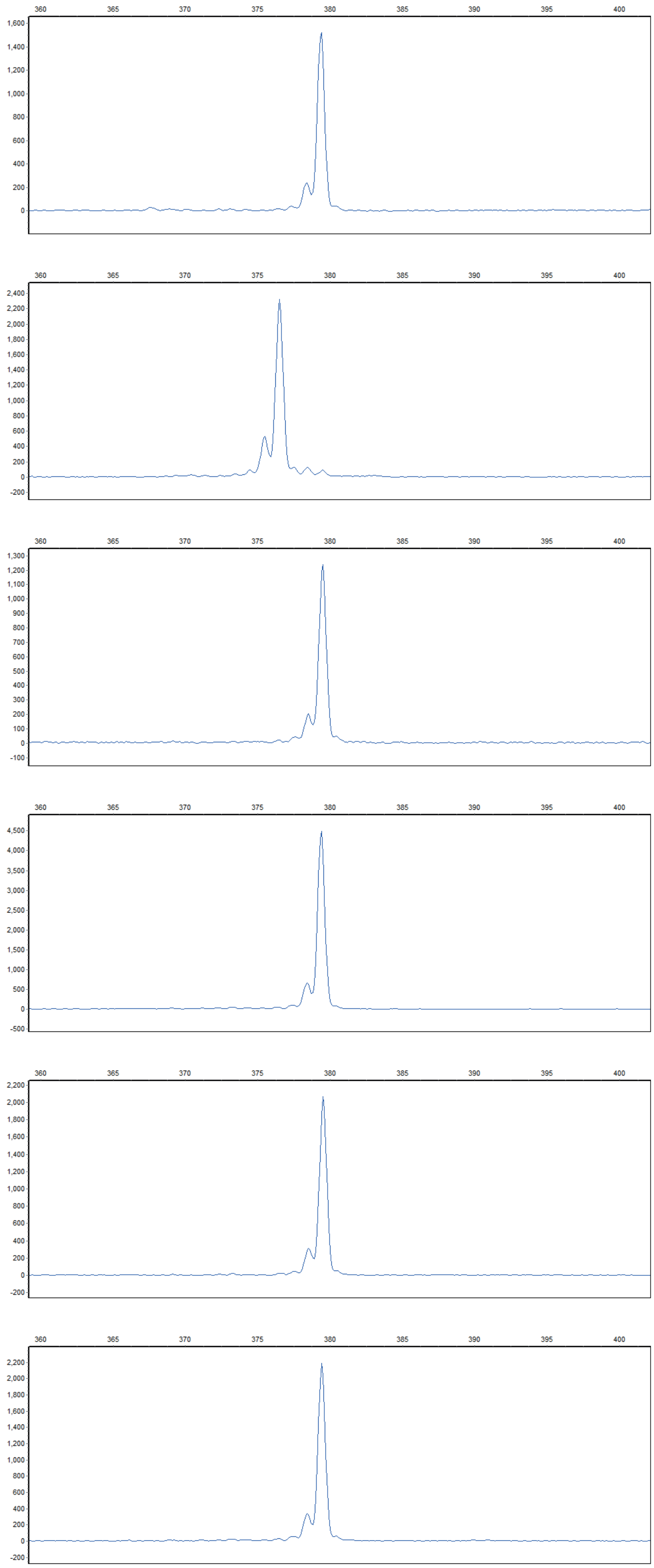

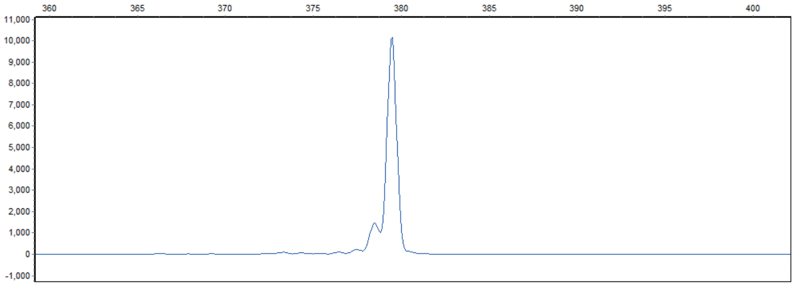
360 365 370 375 380 385 390 395 400
11,000 10,000 9,000 8,000 7,000 6,000 5,000 4,000 3,000 2,000 1,000 0 -1,000

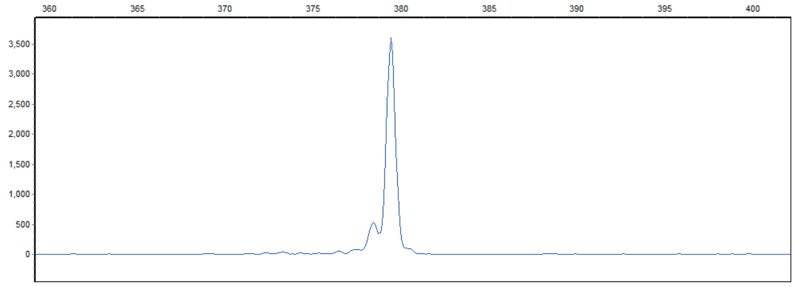
360 365 370 375 380 385 390 395 400
3,500 3,000 2,500 2,000 1,500 1,000 500 0

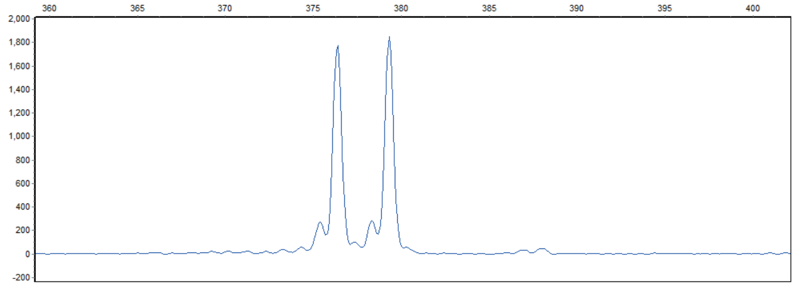
360 365 370 375 380 385 390 395 400
2,000 1,800 1,600 1,400 1,200 1,000 800 600 400 200 0 -200

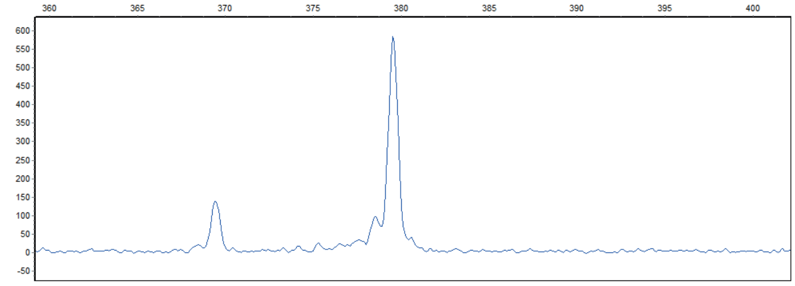
360 365 370 375 380 385 390 395 400
600 550 500 450 400 350 300 250 200 150 100 50 0 -50

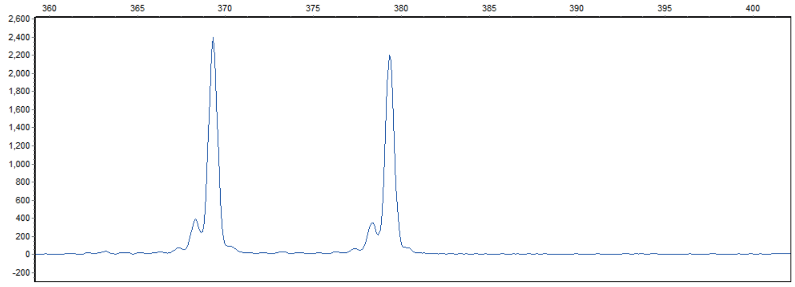
360 365 370 375 380 385 390 395 400
2,600 2,400 2,200 2,000 1,800 1,600 1,400 1,200 1,000 800 600 400 200 0 -200

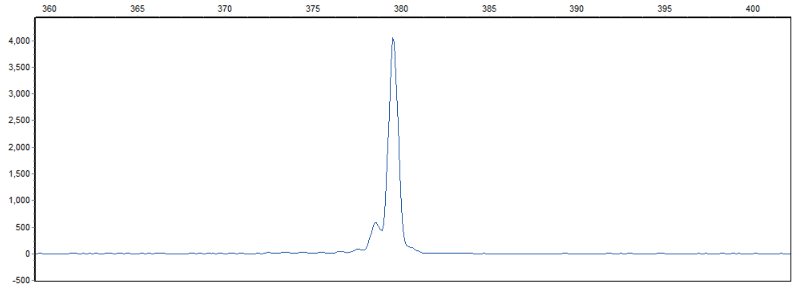
360 365 370 375 380 385 390 395 400
4,000 3,500 3,000 2,500 2,000 1,500 1,000 500 0 -500

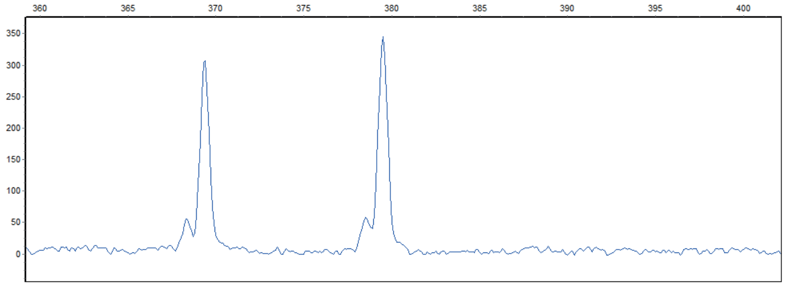
360 365 370 375 380 385 390 395 400
350 300 250 200 150 100 50 0

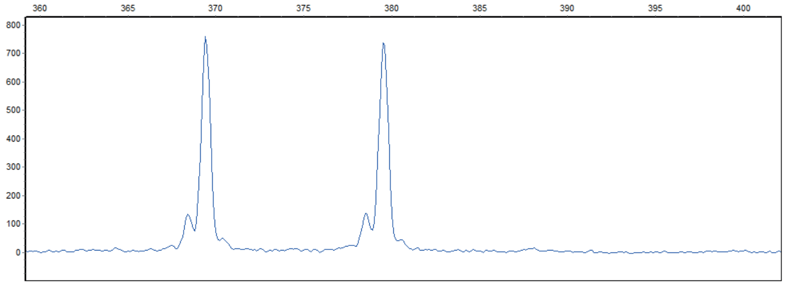
360 365 370 375 380 385 390 395 400
800 700 600 500 400 300 200 100 0

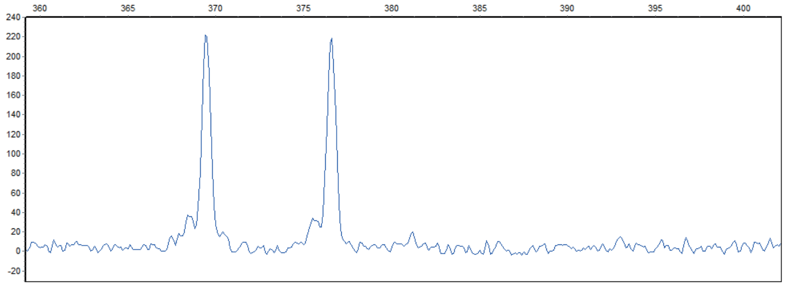
360 365 370 375 380 385 390 395 400
240 220 200 180 160 140 120 100 80 60 40 20 0 -20
